# Immunonutrients involved in the regulation of the inflammatory and oxidative processes: implication for gamete competence

**DOI:** 10.1007/s10815-022-02472-6

**Published:** 2022-03-30

**Authors:** Laura Di Renzo, Antonino De Lorenzo, Marco Fontanari, Paola Gualtieri, Diego Monsignore, Giulia Schifano, Valentina Alfano, Marco Marchetti, On behalf of SIERR

**Affiliations:** 1 Section of Clinical Nutrition and Nutrigenomic, Department of Biomedicine and Prevention, University of Tor Vergata, Via Montpellier 1, 00133 Rome, Italy; 2 School of Specialization in Food Sciences, University of Rome Tor Vergata, Via Montpellier 1, 00133 Rome, Italy; 3 Italian University Network for Sustainable Development (RUS), Food Working Group, University of Tor Vergata, Via Cracovia, 00133 Rome, Italy; 4 PhD School of Applied Medical-Surgical Sciences, University of Rome Tor Vergata, Via Montpellier 1, 00133 Rome, Italy

**Keywords:** Immunonutrients, Supplementation, Infertility, Oxidative stress, Antioxidant system, Inflammation, Gamete competence

## Abstract

**Purpose:**

The purpose of this umbrella review is to bring together the most recent reviews concerning the role of immunonutrients for male and female infertility.

**Methods:**

Regarding immunonutrients and fertility, the authors have analyzed reviews, systematic reviews, and meta-analyses published between 2011 and June 2021. All reviews on animal or in vitro studies were excluded. Relevant keywords to term micronutrients were analyzed alone or in association with other terms such as “gamete competence,” “male OR female fertility,” “male OR female infertility,” “fertile, “folliculogenesis,” “spermatogenesis,” “immunomodulation,” “immune system,” “oxidative stress.”

**Results:**

The primary research has included 108 results, and after screening by title, abstract. and not topic-related, 41 studies have been included by full texts. The results show the molecular mechanisms and the immunonutrients related impact on gamete formation, development. and competence. In particular, this review focused on arginine, glutamine, vitamin C, vitamin D, vitamin E, omega-3, selenium, and zinc.

**Conclusions:**

Inflammation and oxidative stress significantly impact human reproduction. For this reason, immunonutrients may play an important role in the treatment of infertile patients. However, due to the lack of consistent clinical trials, their application is limited. Therefore, the development of clinical trials is necessary to define the correct supplementation, in case of deficiency.

## Introduction

Correct nutrition is an effective way to decrease the risk of occurrence of diseases and the risk factors associated with it. In recent years, numerous studies and reviews have evaluated the role of immunomodulatory diets or their components to enhance recovery from certain diseases [1]. Moreover, nutrition and the respective antioxidant content are involved in diseases that are not related to the immune system.

To date, we know that nutrition plays a key role in the prevention of several diseases and can determine an improvement in health status [2].

A complete diet therapy of all macro- and micronutrients provides the necessary substrates for all cellular activity.

If macro- or micronutrient intake is deficient, supplements can be used to help. Supplements are products that are taken to supplement a balanced diet [3].

In this context, the Mediterranean diet is probably the best dietary pattern to assess adequate intakes of several micronutrients. Indeed, with its typical functional foods, the Mediterranean diet can provide a high intake of micronutrients to improve the nutritional status (NS) and could be helpful in order to prevent or treat micronutrients deficiency [4].

Furthermore, recent studies showed the correlation between the Mediterranean diet and an improvement of several markers related to male (e.g., semen quality) [5] and female (e.g., oocyte quality) infertility [6].

In particular, infertility is related to damaged oocytes and sperm DNA induced by the high level of free-radical molecules [7]. The free-radical formation increases when the antioxidant mechanisms are compromised, due to the diminished concentration of antioxidant enzymes, vitamins, and minerals that play a critical role in the mechanism of various diseases [8].

Free radicals are molecules characterized by the presence of unpaired electrons. This peculiarity gives these elements the ability to react with the surrounding biological molecules, oxidizing them and, often, preventing their normal function. Although reduced concentrations of free radicals are essential in several cellular processes, such as cellular signaling, high concentrations are implicated in several diseases and clinical conditions deviating from the normal state of well-being [9]. Free radicals start the vicious circle that links oxidative stress and chronic inflammation [10].

The development of hypoxic conditions in an increased production of reactive oxygen species (ROS) induces the expression of proinflammatory cytokines and vice-versa [11].

Infertility affects 186 million people globally, 8–12 % of all couples [12].

Beyond genetic issues, a variety of lifestyle choices are implicated in infertility, and in nearly 15–30% of cases, no identifiable cause can be found [13]. However, the etiology of male and female infertility can be linked to oxidative stress, which contributes to chronic inflammation [14]. Furthermore, chronic inflammation affects the reproductive system [7].

The activation of nuclear factor kappa-light-chain-enhancer of activated B cells (NF-κB), by oxidative stress, stimulates the release of cytokines, such as tumor necrosis factor α (TNF-α), interleukin (IL)-6, and IL-1, which are indicators of inflammation, leading to cells’ dysfunction and death.

Thirty to forty percent of impaired fertility is due to female problems. Among the causes of female infertility, there are tubal, uterine, ovarian, and endocrine disorders. In the female germline, the harmful impact of ROS is highlighted, if the exposure is intense and prolonged.

Postovulatory oocytes go into apoptosis and lose their functionality due to a series of events driven by an increase in oxidative stress. Abnormalities in ovarian function have been correlated with an increase in the infiltration of the ovary by macrophages, with a consequent increase in the release of pro-inflammatory cytokine. The inflammation and oxidative stress that occur can compromise the meiotic and cytoplasmic maturation of the oocyte, with a consequent reduction in its evolutionary competence for fertilization and the development of the preimplantation embryo [15].

Moreover, oxidative stress can be caused by, or be the cause of, mitochondrial dysfunction (MD). MD plays an important role in both oocyte maturation and pre-implantation embryo development, leading to abnormalities in meiotic resumption, fertilization, and development of the embryo to the blastocyst stage [9].

Mitochondria are responsible for the production of adenosine triphosphate (ATP) while also generating high amounts of reactive oxygen species (ROS) derived from oxygen metabolism. ROS drive a lipid peroxidation process that culminates in the generation of toxic lipid aldehydes, with a loss of mitochondrial membrane potential, DNA damage, and activation of the intrinsic apoptotic cascade [16].

A vicious cycle is thus generated, between MD which determines a reduction in both the amount of ATP produced and the synthesis of antioxidant molecules and an increase in oxidative stress which in turn increases mitochondrial damage [17].

Environmental stressors (i.e., thermal stress, environmental heat stress, and endocrine-disrupting compounds, EDC, exposure) can cause oocyte developmental competence. Despite the different modes of action of the stressors, the mitochondrial response could be responsible for the reduced quality of the oocytes. Loss of oxidative homeostasis and mitochondrial oxygen free radical (ROS) production might determine the oocyte’s fate [18].

The formation of mitochondrial aggregates in the cytoplasm of oocytes is caused by overexpression of mitochondrial fusion proteins, with a consequence on the organization of the spindle and the distribution of the spacetime endoplasmic reticulum (ER). Moreover, ROS can induce endoplasmic reticulum (ER) stress, correlated to low oocyte quality, causing reduced ovulation, fertilization, and/or pre-implantation development [9].

Furthermore, low-grade chronic inflammation is often associated with several gynecologic disorders associated with female infertility.

Indeed, chronic inflammation plays a key role in the pathophysiology of common gynecological disorders, such as polycystic ovary syndrome (PCOS) [19].

PCOS is a heterogeneous condition associated with an endocrine reproductive disorder. It affects mostly females with ages ranging between 18 and 44 years old [20].

Frequently, PCOS is related to a persistent hormonal disbalance (i.e., androgen excess) linked to the development of numerous cysts ad subsequently to an irregular menstrual cycle, both factors associated with infertility [19].

Yet, PCOS and its typical idiopathic hyperandrogenism are often related to obesity and abdominal adiposity.

The interplay between PCOS and abdominal adiposity may be the result of a vicious circle: androgen excess promotes the abdominal deposition of body fat, and this adipose tissue facilitates androgen excess, which is linked to abnormal insulin production and release, culminating in insulin resistance [21].

Visceral adipose tissue can cause also inflammatory response and the development of chronic low-grade inflammation, resulting in an increased production of inflammatory cytokines and recruitment of the immune cell. Furthermore, the PCOS-altered inflammation processes play a crucial role in folliculogenesis and ovulation. Indeed, the PCOS characteristics can alter the ovarian theca cell morphology, fundamental for oocyte generation, leading to hyperthecosis and oocyte failure.

Moreover, the absence of ovulation makes implantation difficult, and even if this process occurs, complications and abortion risks in pregnancy are often associated with PCOS patients [19].

Among the common gynecologic disorders, endometriosis plays a pivotal role in female infertility which is a leading cause. Classically, endometriosis is defined as the presence of endometrial-type mucosa outside the uterus, mainly in the peritoneal cavity and ovaries. Despite its prevalence, endometriosis remains still understood and without a blood test for its diagnosis [22].

During the last decades, four theories were proposed for endometriosis development (i.e., retrograde menstruation, coelomic metaplasia, and Müllerian remnants).

According to the most convincing model, the retrograde menstruation hypothesis, endometrial fragments reaching the pelvis via transtubal retrograde flow, can implant in the peritoneum and abdominal organs, proliferate, and cause chronic inflammation and estrogen-related symptoms, such as dysmenorrhea, dyspareunia, pain, and infertility. Yet, the excessive inflammation and sex hormone imbalance can alter endometrium receptivity and decidualization, a complex combination of morphological and biochemical changes in the endometrium, fundamental for the correct implantation [23].

Subsequently, TNF-α and IL-16 can sustain the inflammatory processes in the peritoneal cavity through immune cells recruitment, other inflammatory cytokines release. Furthermore, the aberrant ROS production creates a pro-oxidant peritoneal microenvironment, worsening the endothelial functions in women with endometriosis and culminating in a disruption of oocyte maturation and infertility [24].

Low-grade chronic inflammation can also promote tumorigenesis and indicates Endometriosis as a risk factor for ovarian cancer development [25].

The male germline is as vulnerable to oxidative stress as the female one. Numerous evidence suggests that there is a decline in global fertility rates with a parallel decline in sperm counts [26].

There are several recognized causes of impaired sperm parameters. Male infertility may be due to congenital genitourinary abnormalities such as cryptorchidism, acquired genitourinary abnormalities such as vasectomy, malignancy such as in testicular cancer, elevated scrotal temperature as in varicocele, endocrine dysfunction as in hypogonadotropic hypogonadism, genetic abnormalities such as Klinefelter syndrome, and immunologic factors such as anti-sperm antibodies [26]. There are also environmental and behavioral factors that participate in the pathogenesis of the disease, such as radiation, smoking, nutritional deficiencies, electromagnetic waves, air pollution, insecticides, and alcohol [27].

From the analysis of seminal fluid, alterations in concentration, motility, morphology, and combinations of these can be detected, going to configure respectively the conditions of oligozoospermia, asthenospermia, teratozoospermia, and oligoasthenoteratozoospermia [27].

In recent years, it has been observed that even men with sperm parameters in the normal range can present molecular alterations, such as sperm DNA fragmentation (SDF). Furthermore, the poor reproductive capacity of these patients seems to be associated with altered levels of ROS [26].

The presence of ROS, such as superoxide hydroxyl radical and hydrogen peroxide in seminal fluid is of crucial importance to maintain normal reproductive function: vascular tone regulation, gene regulation, sperm capacitation, and acrosome reaction depend all on the precarious balance of the organism redox state [26]. Nonetheless, numerous studies support the role of ROS in male infertility. Excessive levels of ROS that are not balanced by antioxidant systems induce lipid peroxidation, DNA damage, and apoptosis and also generate a state of OS as well [28]. Leukocytes and abnormal sperm are the major sources of ROS in human seminal plasma [29].

It must also be considered that spermatozoa are particularly vulnerable to free radicals, due to the high content of polyunsaturated fatty acids in their membrane and the lack of cytoplasmic antioxidant repair systems [28]. High concentrations of ROS correlate with a worsening of sperm parameters, fertilization rate, embryonic development, and pregnancy rate. These data are also confirmed by the fact that 20–40% of infertile men have higher concentrations of these elements than their healthy counterparts [29]. Therefore, the control of ROS could play a key role in infertility management [27].

Determining a reduction in the level of ROS would be beneficial for sperm function, achieving a range of functions from the cross-linking of sperm chromatin to the enhancement of sperm capacitation.

Therefore, increasing the antioxidant system, through nutrition and supplements, could prevent negative effects on reproduction.

Considering the important role that oxidative stress plays in the etiology of defective sperm and oocyte function, we assume that nutritional strategies may have a therapeutic role to alleviate, to some extent, the effects of stress and inflammation on fertility [30].

The emergent literature on immunonutrients underlines the new fundamental aspect of precision nutrition, able to relate immunity, infection, inflammation, injury, or tissue damage with the individual nutritional status [18].

Immunonutrition is based on the concept that malnutrition impairs inflammatory or immune responses. Therefore, the use of immunonutrients in cases of deficiency can be applied to several cases in which the intake of specific nutrients is needed to modify inflammatory or immune responses [31].

Supplementary administration of specific nutrients is possible to favor the decrease of the state of hyper-inflammation and increase the immune system [13]. Three potential targets exist for immunonutrients: mucosal barrier function, cellular defense, and local or systemic inflammation [31]. Our immune system is based on an innate (or natural) system and an acquired (specific or adaptive) system. The innate immune system includes physical barriers, soluble proteins, and small bioactive molecules such as defensins and ficolins 1-3, pro-inflammatory cytokines, chemokines, lipid mediators of inflammation, membrane-bound receptors, and cytoplasmic proteins. It is also represented by dendritic cells, macrophages, and neutrophils, with phagocytic activity, and by eosinophils, mast cells, and natural killer cells, which release specific soluble antimicrobial factors. The adaptive immune system is represented by B lymphocytes (B cells) and T lymphocytes (T cells), as the cytotoxic T-cells (TC cells), helper T-cells (Th), differentiated into Th1, Th2, and Th17, and suppressor T-cells), natural killer (NK) cells, and NK-T cells. Leukocytes represent the mediator of both innate and adaptative immunity [13]. Th1 differentiation is produced by IL-12 and type I interferon (IFN) or NK cells; Th2 differentiation depends on IL-4 produced by natural killer (NK) 1.1 + T cells, basophils or mast cells; and IL-1b, IL-23, TGFβ, and IL-6 induce differentiation towards Th17 that releases IL-17, IL-22, and antigen-induced regulatory T cell (Treg) [8].

The loss of the self-tolerance system, accompanied by dysfunction of the immune system and the loss of homeostatic equilibrium between TREG cells (interleukin IL-10) and Th17 cells (interleukin IL-17), generates a damaging human tissue, resulting in various diseases [32].

For the correct functioning of the immune system, it is essential to follow a healthy and balanced diet.

Carbohydrates, amino acids, and lipids provide energy to the immune system. Moreover, cells of the immune system can utilize electron carriers and a range of coenzymes, generally vitamins and minerals such as iron (Fe), copper (Cu), and selenium (Se) [33].

The immune system can respond appropriately to different nutrients: Arginine (an essential substrate for an immune cell, in particular for lymphocyte function), glutamine (able to increase T lymphocyte functions), omega-3 (ω-3) fatty acids (exert an anti-inflammatory activity, as a reduction in the synthesis of pro-inflammatory eicosanoids, reduction in leukocyte and platelets-adhesive endothelial interactions, inhibition of inflammatory gene expression by inhibiting NF-κB activity and stimulation of glutathione production which can decrease oxidative injury), nucleotides (derivative compound of purine or pyrimidine, participates in the activation, maturation, and proliferation of lymphocytes, promoting the phagocytic function of macrophages, useful for proper DNA and RNA synthesis), vitamin D, antioxidant compounds (such as vitamin E and C, selenium, copper, iron, and zinc), and mineral (involved in nucleotide and nucleic acid synthesis) [13].

Table 1 shows the characteristic of the most important immunonutrients.

**Table 1:** Characteristics of the immunonutrients under consideration

Nutrient	Sources	RDA	Roles	Reference
L-Arginine	Nuts, seafood, tofu, spinach, seed, brown rice, raisins, coconut, buckwheat, oats, barley cereals, chocolate, dairy products, turkey, pork and beef-	20–30 g/day	Arginine is a precursor to proteins and NO and plays an active role in metabolism, immune function, and the response to infection. It influences the body's oxidative state, DNA repair and cell proliferation. Its metabolism within immune cells is involved in the management of inflammation, pregnancy, and numerous diseases. It enhances the T-cell response by increasing the Th-cell population.	[34, 35]
Glutamine	Animal foods such as meat, fish, eggs, milk, yogurt, and cheese, while vegetable sources include beans, spinach, cabbage, and beetroot (necessarily raw)-	1–1.5 g/day	Glutamine is one of the main energy substrates for the synthesis of purine and pyrimidine nucleotides, NADPH, and antioxidants and is involved in many other biosynthetic pathways involved in the maintenance of cellular integrity and function. It has an important role in controlling the inflammatory response, proliferation, survival, and cell apoptosis. Glutamine also stimulates several metabolic pathways, such as hepatic lipid formation and glycogen synthesis, hepatic and renal gluconeogenesis, and muscle protein synthesis. It has an important role in the maintenance and enhancement of the immune system. Glutamine increases fetal growth and development as well as fetal survival rates. It has a role in preventing the dysfunction of the male reproductive system.	[36, 37]
Vitamin C	Oranges and orange juice, red and green peppers, strawberries, blackcurrants, kiwi, broccoli, brussels sprouts, potatoes.	75–90 mg (+ 85 mg in pregnancy)	↑ collagen synthesis and protects cell membranes from damage caused by free radicals; ↑ keratinocyte differentiation; ↑ lipid synthesis; ↑ fibroblasts proliferation and migration Proliferation, function, and movement of neutrophils, monocytes and phagocytes; ↑ NK cell activities and chemotaxis ↑ Phagocytosis and ROS generation; ↑ microbial killing ↑ Apoptosis and clearance of spent neutrophils from sites of infection by macrophages ↓ Extracellular trap (NET) formation, ↓ tissue damage ↑ Antimicrobial effects; ↑ serum levels of complement proteins Maintains redox homeostasis within cells and protects against ROS and RNS during oxidative burst; regenerates other important antioxidants, such as glutathione and vitamin E, to their active state; modulates cytokine production and ↓ histamine levels Roles in production, differentiation, and proliferation of T cells, particularly cytotoxic T cells; ↑ proliferation of lymphocytes, ↑ generation of antibodies.	[1, 38]
Zinc	Shellfish, meat, cheese, some grains and seeds, cereals, seeded or whole-grain bread.	11–40 mcg (+ 1.3 mg in pregnancy)	Zinc is involved in numerous cellular processes such as maintaining the integrity of skin and mucous membranes, being the cofactor of metalloenzymes. It increases the activity of NK cells and cytotoxic T cells, the development of Tregs cells, the phagocytic capacity of monocytes. It supports immunotolerance and modulates cytokine production, inhibiting the development of a pro-inflammatory phenotype mediated by Th17 and Th19 and influencing the production of IL-2, IL-6, and TNF. It has antioxidant effects.	[35, 38]
Selenium	Brazilian nuts, cereals, beef meat, seafood, eggs.	55 mg (+ 70 mg in pregnancy)	↑ IFN-γ production and immunoglobulins; influencing leukocyte and NK cell function; it is involved as a cofactor in antioxidant systems, signaling, lipid biosynthesis, cell cycle, calcium regulation, and protein folding; GPX play a role in gametogenesis and fertilization in women; GPX4 and selenoprotein P are important for spermatogenesis and for the maintenance of the structural integrity of sperm and organizing chromatin.	[1, 35, 39, 40]
Vitamin D	Oily fish, liver, eggs, fortified foods (spreads and some breakfast cereals).	600–800 U.I.	Promotes intestinal calcium absorption and enterocyte differentiation; facilitates calcium homeostasis and intracellular release for spermatozoa movements; induces RXR binding to VDREs on the nuclear DNA and promotes transcription; ↓ IL-6/IL-8/IL-10 related inflammatory process and modulates IR in PCOS patients.	[41–43]
Omega-3	Fish and other seafood (especially cold-water fatty fish, such as salmon, mackerel, tuna, herring, and sardines), nuts and seeds (such as flaxseed, chia seeds, and walnuts), plant oils (such as flaxseed oil, soybean oil, and canola oil).	300 mg/day	Protect cell membrane from ROS and prevent lipid peroxidation; ↓ PGE2 production by inhibiting the activity of cyclooxygenase-2; stimulate PPAR-γ/RXR nuclear translocation and anti-inflammatory related DNA transcription.	[44–46]
Vitamin E	Vegetable oils, wheat germ, cereals, meat, poultry, eggs, dairy products, fruit, and vegetables.	200–400 mg/day	Protects cell membrane from ROS and prevents lipid peroxidation; ↓ PGE2 production by inhibiting the activity of cyclooxygenase 2; maintains or improves the cytotoxic activity of NK cells; balance between Th1 and Th2; synergy with other antioxidant systems.	[35, 47]

*IFN-y,* interferon-gamma; *NK,* natural killer; *NO,* nitric oxide; *GPX,* glutathione peroxidases; *GPX4,* glutathione peroxidases 4; *ROS,* reactive oxygen species; *PGE2,* prostaglandin E2; *RDA,* recommended daily allowance; *Th1*, T-helper 1; *Th2*, T-helper 2; *TNF*, tumor necrosis factor

To date, there are no data on the role of immunonutrients on gamete competence. Therefore, we hypothesized that a balanced diet could be fundamental to improve gamete competence and possibly only when immunonutrients are deficient could they be considered among the supportive therapies to improve male and female fertility. The purpose of this umbrella review is to summarize the evidence in the literature regarding the link between immunonutrients, oxidative stress, inflammation, and male or female fertility.

This umbrella review tries to answer the question of whether immunonutrient supplementation can be used to improve the competence of male and female gametes.

## Material and method

The literature search was carried out via PubMed and focused on reviews, systematic reviews, and meta-analyses published between 2011 and June 2021 that dealt with the topic of immunonutrients and gamete competence. Only articles written in English and published in peer-reviewed journals were considered. All reviews on animal or in vitro studies were excluded.

Relevant keywords to term micronutrients were analyzed alone or in association with other terms as “gamete competence,” “male OR female fertility,” “male OR female infertility,” “fertile,” “folliculogenesis,” “spermatogenesis,” “immunomodulation,” “immune system,” “oxidative stress.”

The articles were analyzed by four different operators (M.F., D.M., P.G., G.S.). The selection process was carried out by first analyzing the titles, then the abstracts, and finally the full text.

Only articles with information about the individual components were selected, and only those explaining their immunomodulatory effect or their effect on gametes competence.

## Results

**Fig. 1:**
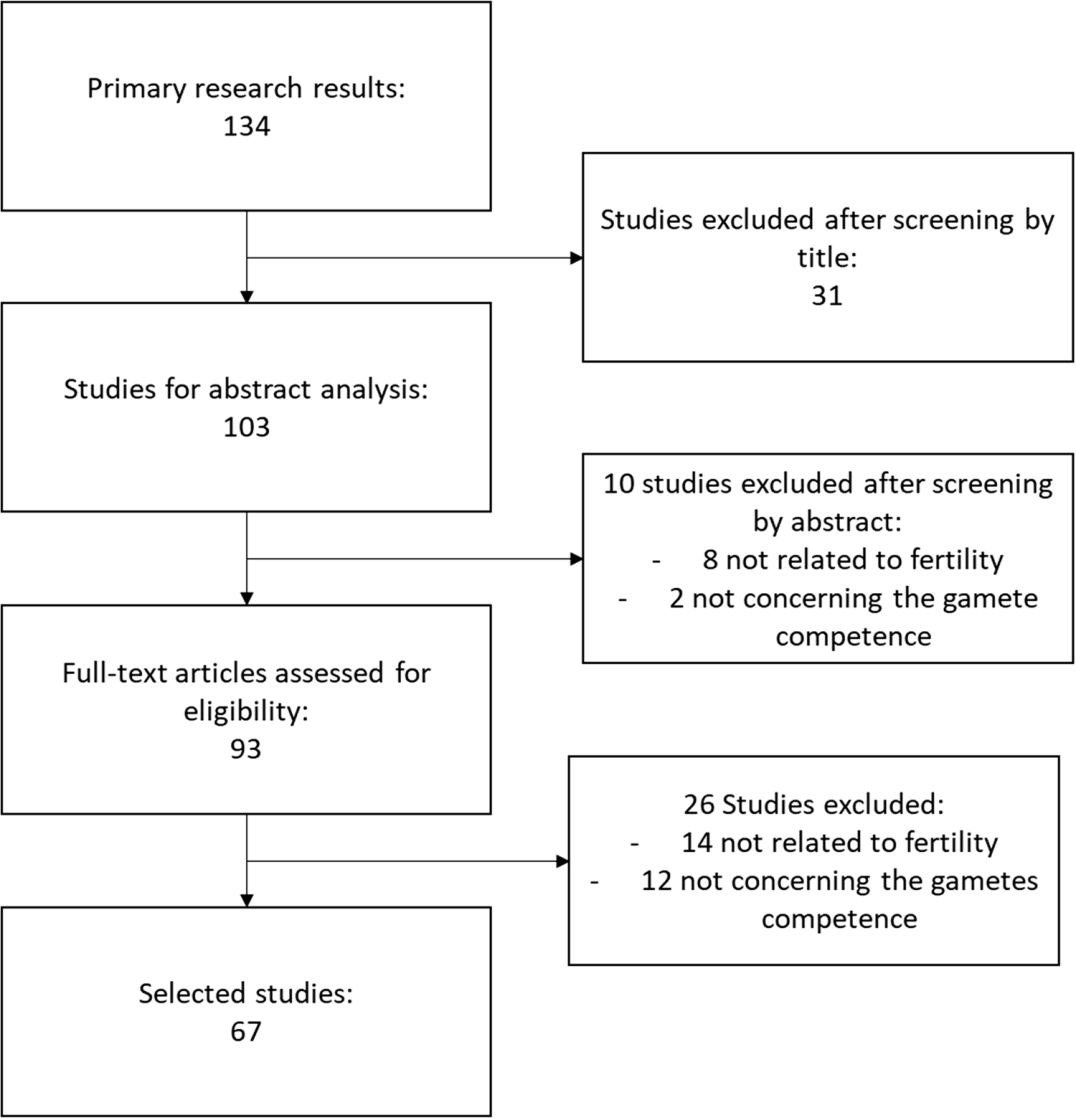
Flowchart of selection of reviews, systematic reviews, and metanalysis published

Following title and abstract screening, 41 papers were excluded and a further 26 after assessing full texts. Therefore, 42 studies were included in the present umbrella review. The figure shows the steps of the selection process (Fig. 1).

Of the 42 articles selected, 7 dealt with arginine, 6 with glutamine, 10 with vitamin C, 11 with vitamin D, 8 with vitamin E, 5 with omega-3, 10 with selenium, and 9 with zinc. The characteristics of the articles included are listed in Table 2.

**Table 2:** Characteristics of included studies

Title	Author	Year	Review type	Included studies	Outcome(s)	Finding(s)
Antioxidant supplements and semen parameters: an evidence-based review.	Ahmadi S, Bashiri R, Ghadiri-Anari A, Nadjarzadeh A.	2016	Review	16 clinical trials	Improving semen parameters such as sperm concentration, motility, morphology, DNA damage, and fertility rate.	Using vitamin C, vitamin E and CoQ10 can improve sperm parameters in infertile men.
A review of the potential interaction of selenium and iodine on placental and child health.	Habibi N, Grieger JA, Bianco-Miotto T.	2020	Review	28 clinical trials on selenium	Correlation between selenium and iodine in pregnancy complications due to oxidative stress.	Micronutrient deficiencies in the preconception period affect the health of the placenta.
Antioxidant therapy in idiopathic oligoasthenoteratozoospermia	Majzoub A, Agarwal A.	2017	Review	21 clinical trials; Cochrane review of 48 randomized controlled clinical trials; 17 randomized trials; 20 trials.	Finding the rationale and evidence supporting the use of antioxidants in idiopathic oligoasthenoteratozoospermia.	Carnitines, glutathione, vitamins E and C, coenzyme-Q10, N-acetylcysteine, selenium, zinc, folic acid, and lycopene reduce sperm damage induced by oxidative stress. Reviews and meta-analyses analyzed reported an improvement in sperm parameters and birth rates.
Antioxidants for female subfertility.	Showell MG, Mackenzie-Proctor R, Jordan V, Hart RJ.	2017	Systematic review	50 Randomized clinical trials	Determine whether oral antioxidant supplement versus placebo, no treatment or standard treatment, or other antioxidants improve fertility outcomes for subfertile women.	Very low-quality evidence shows that taking an antioxidant may provide benefits to subfertile women.
Intracellular signalling during female gametogenesis.	(Sobinoff AP, Sutherland JM, Mclaughlin EA.	2012	Review		The impact of zinc on oocyte development.	A novel role for the metal ion zinc was proposed in the regulation of meiosis I and meiosis II progression through early meiosis inhibitor (Emi2) and Mos-Mapk signaling for the oocyte development.
Changes in vitamin E levels as a marker of female infertility.	Ashraf, M., Mustansir, F., Baqir, S. M., Alam, F., & Rehman, R.	2020	Review		The impact of vitamin E levels of follicular fluid on oocyte competence, embryo development, and pregnancy outcome in patients.	Adequate amount of vitamin E in follicular fluid enhances the possibility of maturation of oocytes.
Diet and fertility: a review	Audrey J. Gaskinks, Sc.D., and Jorge E. Chavarro.	2017	Review		Evaluate the effects of diet and antioxidant compounds on fertility.	Antioxidant compounds can support both male and female fertility.
Diet and nutritional factors in male (in)fertility—underestimated factors.	Kinga Skoracka, Piotr Eder, Liliana Łykowska-Szuber, Agnieszka Dobrowolska and Iwona Krela-Ka´zmierczak.	2020	Review		Evaluate the effects of diet and antioxidant compounds on male infertility and semen quality.	Omega-3 fatty acids, vitamin D, E, zinc, selenium, copper, and manganese supplementation can improve semen quality and male fertility.
Dietary requirements of “nutritionally non-essential amino acids” by animals and humans.	Wu, G., Wu, Z., Dai, Z. et al.	2012	Review		Needs of experimental evidence to support the assumption of non-essential amino acids.	Glutamine acts a role in gene expression, cell signaling, antioxidative responses, neurotransmission, and immunity. In addition, glutamine is among the main metabolic fuels of the small intestine in maintaining both digestive function and protecting the integrity of the mucosa.
Dietary fatty acids affect semen quality: a review.	V. Esmaeili, A. H. Shahverdi, M. H. Moghadasian and A. R. Alizadeh.	2015	Review	11 clinical trials	Evaluate the effects of Omega-3 fatty acids on semen quality.	Omega-3 fatty acids supplementation can ameliorate the concentration of spermatozoa, percentages of progressively motile and morphologically normal spermatozoa.
Dietary supplements for male infertility: a critical evaluation of their composition.	Garolla A, Petre GC, Francini-Pesenti F, De Toni L, Vitagliano A, Di Nisio A, Foresta C.	2020	Review		Critical analysis of dietary supplements.	There are nutritional supplements on the Italian market consisting of nutrients with proven efficacy, but also of nutrients whose benefits have not been scientifically proven and which may even be harmful.
Effectiveness of omega-3 fatty acid for polycystic ovary syndrome: a systematic review and meta-analysis.	Kailin Yang, Liuting Zeng , Tingting Bao and Jinwen Ge.	2018	Review	15 clinical trials	Evaluate the effects of omega-3 fatty acids in PCOS-related infertility.	Omega-3 fatty acids acid may be recommended for the treatment of PCOS with insulin resistance.
Effects of zinc deficiency on impaired spermatogenesis and male infertility: the role of oxidative stress, inflammation, and apoptosis.	Beigi Harchegani A, Dahan H, Tahmasbpour E, Bakhtiari Kaboutaraki H, Shahriary A.	2020	Review		To discuss the molecular mechanisms by which zinc is involved in male reproduction.	Zinc deficiency is implicated in spermatogenesis disorders and male infertility. It induces oxidative stress with consequent damage to Leydig cells, alteration of steroidogenesis, accumulation of leucocytes, and inflammation. It may be useful to screen all infertile men for this defect.
Empirical medical therapy in idiopathic male infertility: promise or panacea?	Jung JH, Seo JT.	2014	Review		Evaluate the literature regarding the most common empirical medical treatments (hormonal treatment and antioxidant supplementation) in idiopathic male infertility.	Empirical medical treatments appear to have a positive effect on male fertility, but standardized protocols need to be established in order to implement them effectively.
Evidence for a manifold role of selenium in infertility.	Mintziori G, Mousiolis A, Duntas LH, Goulis DG.	2019	Review		Evaluate the role of selenium in male and female infertility.	Studies are needed to prove the benefits of Selenium supplementation in men and women.
Glutamine: metabolism and immune function, supplementation and clinical translation.	Cruzat V, Rogero M.M, Keane K.N, Curi R, Newsholme P.	2018	Review		Role of glutamine in cells of the immune system; glutamine metabolism and action.	Immune cells and their functions are largely dependent on the availability of glutamine. During catabolic/hypercatabolic situations glutamine can become essential for metabolic function.
Immunological role of vitamin D at the maternal-fetal interface.	Tamblyn JA, Hewison M, Wagner CL, Bulmer JN, Kilby MD.		Review		Evaluate the effects of vitamin D during pregnancy.	Gain of responses and modulation of T-lymphocytes to suppress inflammation and promote tolerance mediated through vitamin D actions.
Impact of arginine nutrition and metabolism during pregnancy on offspring outcomes.	Hsu CN, Tain YL.	2019	Review		Analysis of the influence of arginine during pregnancy, the consequences of its deficiency, and its role in the prevention of NCDs.	Arginine plays an important role both during pregnancy and in the long-term health of the unborn child. However, in order to best influence the metabolic pathways of this amino acid further studies are needed Currently, the evidence supporting the role of micronutrients and vitamins in the prevention of pre-eclampsia is not strong enough to justify their systematic use.
Interactions between estrogen and 1α,25(OH)2-vitamin D3 signalling and their roles in spermatogenesis and spermatozoa functions.	Ana Paula Zanatta, Vanessa Brouard , Camille Gautier , Renata Goncalves, Hélène Bouraïma-Lelong , Fátima Regina Mena Barreto Silva and Christelle Delalande.	2017	Review		Understanding molecular mechanism and signaling pathways of VD in spermatogenesis.	VD plays a key role in spermatogenesis through genomic- and non-genomic pathways.
Monosodium glutamate (MSG)-induced male reproductive dysfunction: a mini review.	Kayode OT, Rotimi DE, Kayode A, Olaolu TD, Adeyemi OS.	2020	Review		Monosodium glutamate (MSG) has been found to be potent in articulating reproductive abnormalities in males.	The different mechanisms involved include spermatogenic alteration, histological alteration, and hormonal imbalances.
MOSH syndrome (male obesity secondary hypogonadism): clinical assessment and possible therapeutic approaches.	Antonino De Lorenzo, Annalisa Noce, Eleonora Moriconi, Tiziana Rampello,Giulia Marrone, Nicola Di Daniele,and Valentina Rovella.	2018	Review		Potential role of omega-3 fatty acids in male obesity secondary hypogonadism.	Omega-3 fatty acids supplementation can support the restore of the balanced male hormone axis.
Multiple micronutrient supplementation and birth outcomes: the potential importance of selenium.	Perkins AV, Vanderlelie JJ.	2016	Review		Examine the use of micronutrients during pregnancy and current recommendations for these products.	A beneficial effect in pregnancy has been shown e reduce the risk of preeclampsia and preterm labor, especially in overweight and obese women.
Novel insights on the role of nitric oxide in the ovary: a review of the literature.	Budani MC, Tiboni GM.	2021	Review		Analysis of data on the role of arginine at the ovarian level.	Arginine plays an important role in steroidogenesis and folliculogenesis, as well as being crucial in oocyte competence.
Nutrient supplementation: improving male fertility fourfold.	Mora-Esteves C, Shin D.	2013	Review	34 randomized controlled trials	Evaluate the improvement of outcomes of assisted reproduction and the action of different types of antioxidants.	A definitive conclusion cannot be drawn because of the heterogeneous literature.
Nutritional approach to preeclampsia prevention.	Achamrah N, Ditisheim A.	2018	Review		To analyze the latest data on the nutritional approach to pre-eclampsia.	Currently, the evidence supporting the role of micronutrients and vitamins in the prevention of pre-eclampsia is not strong enough to justify their systematic use.
Regulation of protein metabolism by glutamine: implications for nutrition and health.	Xi P, Jiang Z, Zheng C, Yingcai , Wu G.	2011	review		Evaluation of the utility of glutamine supplementation in enteral diets or parenteral solutions to improve nitrogen balance with glutamine deficiency.	Glutamine could stimulate protein synthesis and inhibit proteolysis in skeletal muscle. glutamine concentrations show marked reductions in response to infection, sepsis, severe burns, cancer, and other pathological factors.
Role of dietary amino acids and nutrient sensing system in pregnancy associated disorders.	Hussain T, Tan B, Murtaza G, Metwally E, Yang H, Kalhoro MS, Kalhoro DH, Chughtai MI, Yin Y.	2020	Review		Exploring the importance of dietary amino acids and their metabolic pathways on pregnancy.	Amino acid supplementation has a positive impact on fertility, improving many aspects of pregnancy and fetal life.
Role of oxidative stress in female reproduction.	Agarwal A, Gupta S, Sharma RK.	2005	Review		Role of nitric oxide species in female reproduction, interaction between cytokines and oxidative stress in the etiology of female reproductive disorders.	Combination of intervention strategy of vitamin E and vitamin C supplementation in preventing preeclampsia are highlighted. Antioxidants are powerful and there are few trials investigating antioxidant supplementation in female reproduction. However, randomized controlled trials with sufficient power are necessary to prove the efficacy.
Role of selenium and selenoproteins in male reproductive function: a review of past and present evidences.	Qazi IH, Angel C, Yang H, et al.	2019	Review	8 human studies	Evaluation of selenoproteins in male reproduction.	No definitive conclusions on their effects were identified.
Selenium, selenoproteins, and female reproduction: a review.	Qazi IH, Angel C, Yang H, et al.	2018	Review		Biological functions of Selenium and selenoproteins and the relationship between Selenium and female reproductive function.	Studies not yet sufficient to draw valid conclusions.
Strengthening the immunity of the Swiss population with micronutrients: a narrative review and call for action.	Berger MM, Herter-Aeberli I, Zimmermann MB, Spieldenner J, Eggersdorfer M.	2021	Review		Evaluate the effects of micronutrients, such as Vitamin D, Omega-3 FAs, Vitamin C, Iron for optimal immune function and prevention of respiratory tract infections.	Strong relationships between micronutrient and n-3 PUFA status and immune function.
Systematic review of antioxidant types and doses in male infertility: Benefits on semen parameters, advanced sperm function, assisted reproduction and live-birth rate.	Majzoub A, Agarwal A.	2018	Systematic review	19 randomized clinical trials; 10 prospective studies	Evaluation of the effect on male fertility of oral antioxidant supplementation.	26 studies showed a significant effect on baseline semen parameters, advanced sperm function, outcomes of assisted reproductive therapy, and birth rate.
The excessive use of antioxidant therapy: A possible cause of male infertility?	Henkel R, Sandhu IS, Agarwal A.	2019	Review	8 clinical trials	Establish the presence of benefits and risks of antioxidant therapy for male infertility.	The use of antioxidants must be considered with caution as in addition to the beneficial effects they have side effects.
The role of over-the-counter supplements for the treatment of male infertility--fact or fiction?	Ko EY, Sabanegh ES Jr.	2012	Review	22 clinical trials	Evaluate the effects of micronutrients on male fertility.	No definitive conclusions on their effects were identified and the dosage could not be identified.
The role of selenium in human conception and pregnancy.	Pieczyńska J, Grajeta H.	2015	Review	11 clinical trials	Demonstrate the correlations with problems related to procreation.	Selenium supplementation in case of deficiencies is important, both for women and men.
Vitamin C and immune function.	Carr A.C, Maggini S.	2017	Review		Role of vitamin c in the modulation of the immune system.	Vitamin C has a potent antioxidant role against ROS; its action as a cofactor for numerous biosynthetic and gene regulatory enzymes plays a key role in its immunomodulatory effects.
Vitamin C as an antioxidant supplement in women's health: a myth in need of urgent burial.	Talaulikar VS, Manyonda IT.	2011	Review		Vitamin supplements with antioxidant properties, such as vitamins C, could also prevent pre-eclampsia and improve pregnancy health.	Despite the role of oxidative stress in the pathophysiology of pre-eclampsia and the possibility of improvement of the disease by an antioxidant administered at the right time and in the correct dosage, there is no strong evidence for great benefits.
Vitamin D and aspects of female fertility.	Nick Voulgaris, Labrini Papanastasiou, George Piaditis, Anna Angelousi, Gregory Kaltsas, George Mastorakos, Eva Kassi.	2017	Review	15 clinical trials	Evaluate the effects of VD supplementation in PCOS, Endometriosis related female infertility, and for IVF and implantation.	VD can support female fertility in PCOS, endometriosis, and IVF.
Vitamin D and health - the missing vitamin in humans.	Chang SW, Lee HC.	2019	Review		Determine the lacking effects of Vitamin D on human health.	Appropriate vitamin D supplementation is recommended to obtain optimal plasma concentration.
Vitamin D and obesity: two interacting players in the field of infertility.	Julia K. Bosdou, Eirini Konstantinidou, Panagiotis Anagnostis, Efstratios M. Kolibianakis, and Dimitrios G. Goulis.	2019	Review	40 clinical trials	Evaluate the effects of VD supplementation in male infertile subjects.	VD can support spermatogenesis and male fertility in both healthy and obese men.
Vitamin D in endometriosis: a causative or confounding factor?	Sayegh L, Fuleihan Gel-H, Nassar AH.	2014	Review	6 clinical trials	Evaluate the effects of VD in endometriosis-related infertility.	VD can acts as an immunomodulator and anti-inflammatory agent, in the pathogenesis and treatment of endometriosis.
Vitamin D metabolism and guidelines for vitamin D supplementation.	Ramasamy I.	2020	Review		Define the proper VD supplementation and determine the correct serological values for VD.	Supplementation of VD should be included from 400 to 1000 IU/d (10–25 μg/day)
Vitamin D and its role during pregnancy in attaining optimal health of mother and fetus.	Wagner CL, Taylor SN, Dawodu A, Johnson DD, Hollis BW.	2012	Review	2 clinical trials	Overview of vitamin metabolism, states of deficiency.	Vitamin D supplementation is necessary to achieve an optimal 1,25(OH)D3 levels in pregnancy.

*DNA*, deoxyribonucleic acid; *PCOS*, polycystic ovary syndrome; *NCD*, non-communicable disorders; *VD*, vitamin D; *IVF*, in vitro fertilization

Regarding studies examining the role of arginine, Hsu and Tain focused on the metabolism and catabolism of arginine and its effects on all stages of pregnancy [48]. Three reviews analyzed the antioxidant effect of arginine and its impact on fertility [1, 38, 49]. In their review, Budani and Tiboni focused on NO, an arginine catabolite, and its effects on steroidogenesis, folliculogenesis, and gamete competence [50].

In three studies, reference is made to the positive effects of an arginine supplement on male fertility [1, 34, 51].

Recent studies have investigated the role of glutamine in activating genes that control the inflammatory response, metabolism, cell proliferation, survival, and apoptosis. Glutamine is critical in fetal growth, development, and survival by stimulating molecular signaling pathways such as the (mTOR) [52]. Hussain T et al in a recent review studied the role of dietary amino acids and nutrient-sensing system in pregnancy-associated disorders [48]. Glutathione, a high antioxidant molecule that is also produced from glutamine, is important for male and female fertility. It can improve sperm health and quality. Finally, Kayode et al., in a 2020 paper, showed how monosodium glutamate (MSG), the sodium salt of glutamic acid, a common food additive, can induce male reproductive dysfunction [53].

Regarding vitamin C, in a Cochrane review by Mora-Esteves and Shin D, emphasis was placed on the fact that high concentrations of vitamin C have a potent immunomodulatory power on male fertility due to its antioxidant activity [1]. In addition, a combination with vitamin E has been proposed for ROS scavenging action in human sperm. Vitamin C can effectively scavenge ROS also in female gametes and plays a crucial role in both healthy fertility and infertility-related dysfunction [47]. In addition, several clinical studies, including that of Showell and colleagues, have examined human tolerance and efficacy on oocyte quality, follicle number, and estradiol levels through vitamin C supplementation [38].

Regarding vitamin D, two reviews focused on the estrogen-related effects on female gametes through the classic ESR1/ESR2 related signaling pathway [54, 55]. Moreover, Voulgaris et al. suggest the relationship between vitamin D and RXR associated DNA transcription, necessary for the correct development and maturation of the oocytes [41]. Concerning male fertility, Zanatta et al. [42] and Bosdau et al. [56] focused their attention on the hormone balance, estrogen-related pathways, and supplementation for the correct spermatogenesis and the hormone axis equilibrium.

Among the studies considering vitamin E, a review focused particularly on the role of this vitamin in the treatment of oligoasthenoteratozoospermia [28]. Another review focused on the antioxidant activity of this vitamin and highlighted the adverse events due to its excessive dosage [57].

Two articles focused on semen parameters and on how the administration of vitamin E, together with other nutrients, has been observed to reduce sperm DNA damage and improve sperm concentration, motility, and morphology, also leading to better birth rates and assisted reproductive outcomes [27, 58]. Another review focused on the effect of administering various nutrients, including vitamin E, on fertility, although with mixed results [34].

Furthermore, analyzing the effects of omega-3 in the field of fertility, Esmaeili et al. expressed in their review the key role of these micronutrients in the antioxidant and anti-inflammatory pathways driven by PPAR-γ and their related effects on semen quality [44], whereas Noce et al. proposed an interesting supplementary treatment based on omega-3 for the male obesity secondary hypogonadism [45]. Instead, for female fertility, Gaskins et al. theorized the impact of omega-3 in PCOS-associated infertility, closely related to insulin resistance [59]. Yang et al. based their study on the relationship between omega-3 and PCOS-related infertility, with secondary outcomes such as evaluating HOMA index and plasma levels of total cholesterol, triglycerides, and adiponectin [46].

Among the studies concerning selenium, three reviews examined recommendations on the use of supplements in women, and how selenium and selenoproteins deficiencies may affect the protection of mitochondria from oxidative stress [39, 60, 61]. In two reviews the role of selenium in both the male and female reproductive system was highlighted [40, 62]. Two other articles highlighted how various types of antioxidants (in particular selenium and vitamin E,) have had variable efficacy in improving male infertility [1, 63].

Among the eight reviews reporting data about zinc, five focus on the role of this element on spermatogenesis, testicular development and function, sex hormone synthesis, and the control of inflammation and OS in humans [1, 29, 34, 43, 64]. In “Nutritional approach to preeclampsia prevention,” the authors consider the role of zinc on pre-eclampsia, but also refer to its functions within the body and recommended doses in pregnancy [65]. Four reviews report data about the effect of zinc deficiency and supplementation on sperm parameters [27, 28, 34, 58].

**Fig. 2:**
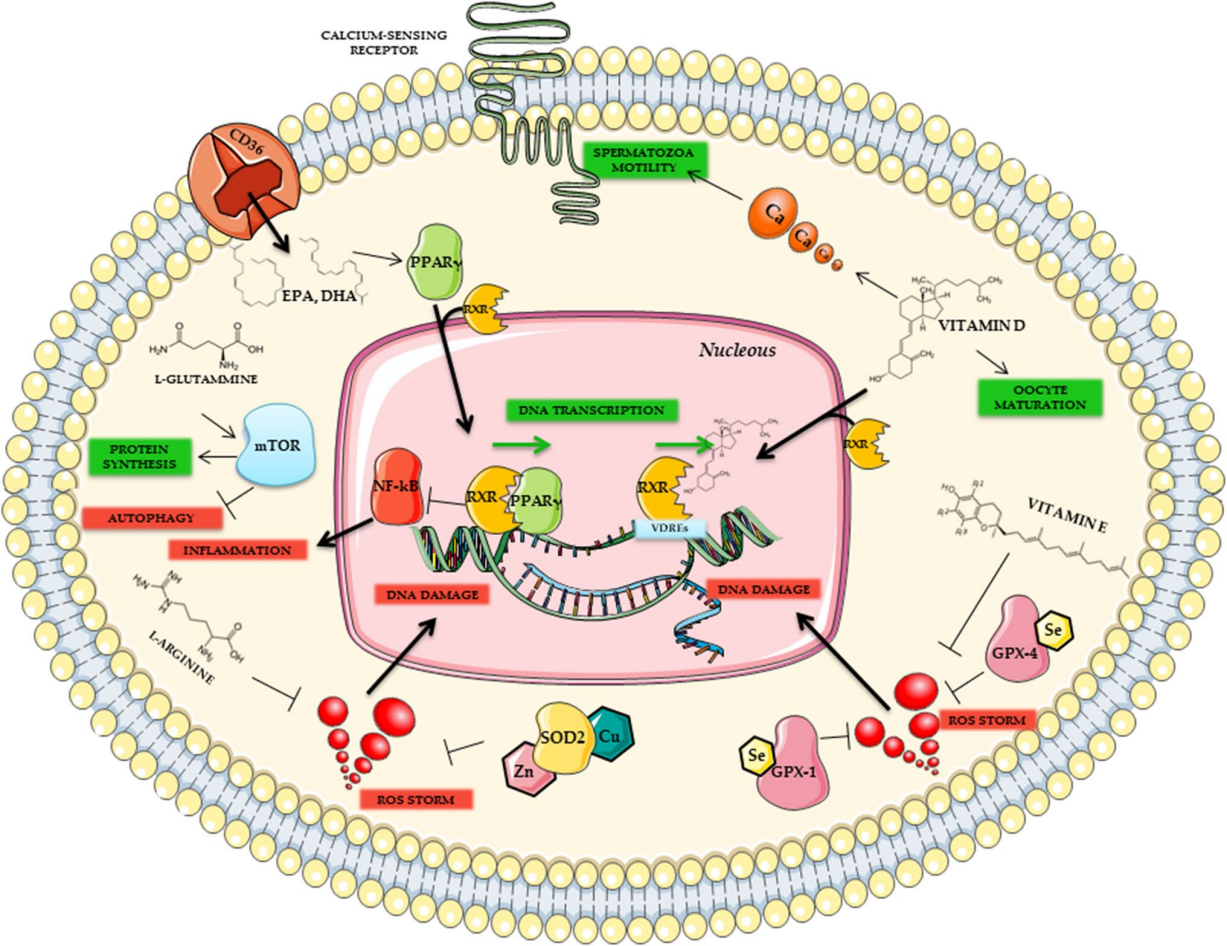
Main signaling pathways in male and female gametes. Omega-3 (ω-3) fatty acids (EPA, DHA) can promote the heterodimer peroxisome proliferator-activated receptor-γ/retinoid X receptor (PPAR-γ/RXR) activation and migration through the nuclear membrane to enhance DNA transcription, blocking Nuclear factor kappa-light-chain-enhancer of activated B cells (NF-κB) related inflammation processes. Vitamin D (VD) can lead RXR related DNA transcription via vitamin D responsive elements (VDREs) binding, essential for correct spermatogenesis and oocytes formation/development. In addition, VD can increase intracellular calcium (Ca^2+^) necessary for spermatozoa motility or oocytes maturation. Selenium (Se), as an essential cofactor for Glutathione peroxidase (GPx) enzymes and vitamin E, can lock reactive oxygen species (ROS) storm from lipid peroxidation. Zinc (Zn) binding to superoxide dismutase 2 (SOD2) can block intracellular ROS production, assisted by L-Arginine activity through inhibition of nitric oxide (NO) production. Activation of mammalian target of rapamycin (mTOR) by L-glutamine can inhibit autophagy and promote protein synthesis

Figure 2 illustrates the main signaling pathways in both male and female gametes.

## Discussion

This umbrella review wants to emphasize the relationship between immunonutrients, oxidative stress, inflammation, and male or female fertility.

### L-Arginine

Arginine is a semi-essential amino acid found in numerous molecules in our bodies. Although we get a certain amount from the diet, 15% is synthesized from citrulline and 80% is recovered from protein turnover [21]. Arginine can be found in nuts, seafood, tofu, spinach, seed, brown rice, raisins, coconut, buckwheat, oats, barley cereals, chocolate, dairy products, turkey, pork, and beef [34]. The recommended daily dose is 20–30g [50].

This amino acid directly stimulates protein synthesis via the mTOR pathway and is essential for the elimination of ammonia from the blood, which at high levels would cause an increase in pH and oxidative stress. Moreover, arginine is a precursor to polyamines (putrescine, spermine, and spermidine), which are involved in DNA replication and cell division [50].

#### The role of L-arginine for female gamete competence

Although less than 2% of arginine is converted to NO and polyamines, these two compounds are crucial in all steps of pregnancy, from fertilization to implantation, and are involved in protein synthesis. In the ovaries, NO is produced by ovarian cells, vessels, and macrophages and has numerous effects. Studies have shown an inhibitory role in the secretion of estradiol and progesterone, as the amino acid inhibits the aromatase enzyme directly and indirectly. Other studies have shown that administration of a NO donor causes a decrease in estradiol but a concomitant increase in progesterone synthesis. At the folliculogenesis level, NO would have a pro- or anti-apoptotic effect depending on its concentration. Many authors, however, based on animal data, suggest that NO has an anti-apoptotic role linked to inhibition of the Fas-FasL system [50].

In addition, NO appears to have a cytostatic effect on immature follicles, and the presence of its inducible synthetase is crucial for follicles to remain dormant. Finally, nitric oxide is implicated in the modulation of the oocyte meiotic cycle, probably by a concentration-dependent mechanism [50]. No reference could be found on the importance of arginine supplementation on gamete competence. However, its supplementation in pregnant women would appear to have a therapeutic effect on intrauterine growth restriction (IUGR) [49]. Despite this, L-arginine supplementation, when compared with a placebo, showed no significant effect on the clinical pregnancy rates [38].

#### The role of L-arginine for male gamete competence

Studies have shown that arginine supplementation in males may improve sperm parameters, particularly concentration and motility. Indeed, NO and spermine are essential for sperm motility. However, the literature is lacking on the subject and further studies are needed [51].

### Glutamine

Glutamine is a neutral non-essential amino acid. Glutamine is the most abundant and versatile amino acid in the body; its endogenous production is between 40 and 80 g/day and its concentration is highest in the liver and skeletal muscle, even more so than in plasma. Glutamine is one of the main energy substrates for rapidly dividing cells. It is of fundamental importance for cell metabolism, nitrogen exchange via ammonia (NH3), transport between tissues, and pH homeostasis: the formation of ammonia from glutamine is vital for renal regulation of acid-base balance. In almost all cells, glutamine can be used as a substrate for the synthesis of purine and pyrimidine nucleotides essential for cell proliferation, NADPH, antioxidants, and many other biosynthetic pathways involved in maintaining cellular integrity and function. Glutamine regulates the expression of several genes involved in cell metabolism, signal transduction, cellular defense, regulation of cellular repair, and activation of intracellular signaling pathways. The action of glutamine also involves the activation of signaling pathways by phosphorylation, such as NF-κB and MAPK [36]. Recent studies have investigated the role of glutamine in activating control genes in the inflammatory response, metabolism, cell proliferation, survival, and apoptosis. Changes in extracellular glutamine concentrations determine the activation of several signaling pathways (ERK, JNK, PKA, and mTOR) and several transcription factors including bZIP proteins (ATF, C/EBP), helix-turn-helix proteins (HSF-1), zinc fingers proteins (Sp1), and nuclear receptors (PPAR, FXR/ RXR). The anti-inflammatory role of glutamine has been extensively studied both in vivo and in vitro and there is strong evidence of its action in regulating cytokine production and the involvement of specific transcription factors, mainly NF-κB. A protective effect of glutamine in the diseased gut has also been demonstrated through inhibition of the DNA-binding activity of AP-1. This was mediated by stimulation of peroxisome proliferator-activated receptor c (PPAR-c) and through a decrease in the phosphorylated form of STAT1 and STAT5. In addition, glutamine modulates the activity of transcription factors at several levels, in the synthesis, post-transcriptional modifications, degradation, or modulation of activating or inhibiting factors. In parallel with its role as a metabolic substrate, glutamine also stimulates a number of metabolic pathways, such as hepatic lipid formation and glycogen synthesis, hepatic and renal gluconeogenesis, and muscle protein synthesis [52]. Immune cells are largely dependent on the availability of glutamine to survive, proliferate and function, and ultimately defend our bodies from pathogens. Glutamine acts as an energy substrate for leukocytes and plays an essential role in cell proliferation, tissue repair processes, and intracellular pathways associated with pathogen recognition. Glutamine is an important modulator of leukocyte function, in lymphocytes, neutrophils, and macrophages. Glutamine depletion reduces lymphocyte proliferation, impairs the expression of surface activation proteins and cytokine production, and induces apoptosis in these cells. Glutamine, via glutamate, together with cysteine and glycine is the precursor amino acid for the synthesis of glutathione (GSH), an antioxidant that can react directly with ROS in oxidative stress, generating oxidized GSH. However, of these three amino acids, glutamate is the first and most important in the synthesis of GSH intermediates. Glutamate synthesis, in turn, depends on the intracellular availability of glutamine. Therefore, a higher glutamine/glutamate ratio enhances the availability of precursors for GSH synthesis. In acute inflammatory situations, such as sepsis or viral infection, there is an increase in the intracellular redox state and all cellular compartments are vulnerable to oxidative stress.

#### 2a.The role of glutamine for female gamete competence

During pregnancy, it is well known that essential amino acids play an important role in optimal fetal growth and in maintaining all the functions essential for successful gestation. However, an indispensable role is played by all the so-called non-essential amino acids, such as glutamine, which is crucial in fetal growth, development, and survival by stimulating certain molecular signaling pathways such as the rapamycin pathway (mTOR), a protein kinase that regulates intracellular protein synthesis. It plays a central role in protein synthesis in the placenta, uterus, and fetus by inhibiting proteolysis in skeletal muscle [49]. Non-essential amino acids are far from being non-essential as they are equally important in the regulation of gene expression, cell signaling, neurotransmission and immunity, growth, lactation, reproduction, and responses against oxidative stress that causes disorders in pregnancy, such as pre-eclampsia, pre-term delivery, and low weight for gestational age. Dietary supplementation of L-glutamine together with L-alanine has been shown to increase fetal growth and development, as well as fetal survival rates by reducing the effects of virus infection. Dietary supplementation of glutamine in this context is crucial because it increases intestinal expression of genes required for cell growth and removal of oxidants, while reducing the expression of genes that promote oxidative stress and immune activation. Glutamine improves protein synthesis in both skeletal muscle and the small intestine [66]. Low birth weight preterm infants have been shown to be glutamine deficient. In addition, these infants have a high rate of protein degradation. Glutamine supplementation resulted in an increase in plasma levels of this amino acid and inhibited protein degradation but did not lead to increased protein or growth in the infant. It is also possible that a deficiency of other amino acids such as arginine limits the beneficial effect of glutamine because arginine is a major factor regulating muscle protein synthesis in infants. However, glutamine supplementation has decreased infections, morbidity, and hospital costs.

Glutamate and glutamine on the other hand are precursors for arginine synthesis. Since there is an increased need for them during fetal growth, they are considered conditionally essential during pregnancy. The molecule N-carbamoyl glutamate (NCG) can activate carbamoylphosphate synthase, which is a key enzyme in the process of arginine synthesis in enterocytes from carbamoyl phosphate and ornithine. Supplementation of NCG during pregnancy has been shown to improve IUGR in animal studies on ruminants as NCG was not affected by metabolic degradation. However, the long-term effects of glutamate and glutamine on offspring health are still under investigation [48]. Glutamate can replace glutamine for many functions such as ATP production, arginine, and glutathione synthesis in small intestinal epithelial cells. In addition, glutamate inhibits glutamine degradation by mitochondrial phosphate-dependent GLS in extrahepatic tissues and cells by increasing cellular glutamine availability. However, some glutamine functions (e.g., glucosamine synthesis, nucleotide synthesis, mTOR activation, and regulation of ornithine decarboxylase expression) cannot be performed by glutamate. Furthermore, although the small intestine catabolizes both glutamine and glutamate from the diet, the intestine only takes glutamine from the circulation [37].

#### The role of glutamine for male gamete competence

As for glutamate, monosodium glutamate (MSG), which is found in several foods, including beef, milk, tuna, and vegetables, plays an important role in human metabolism. MSG has also been associated with male reproductive dysfunction. The various mechanisms by which it can induce reproductive dysfunction include altered sperm production in number and alterations in the process of spermatogenesis with elevated sperm count and decreased pH of the seminal fluid. The changes also affect histology with evidence of testicular hemorrhages, distorted germ cells, and few Sertoli cells. In addition, studies have documented a decrease in the weight of the testis, epididymis, seminal vesicle, and prostate. Hormonal balance is also involved with an imbalance of gonadotropins (reduced concentrations of testosterone, luteinizing hormone, and follicle-stimulating hormone). Underlying this is evidence of oxidative damage with increased lipid peroxidation and reduced activity of antioxidant enzymes [53].

### Vitamin C

Vitamin C is an essential micronutrient for humans, with pleiotropic functions related to its ability to donate electrons. Humans are unable to synthesize vitamin C, so it is strictly obtained through the dietary intake of fruits and vegetables. Citrus fruits, berries, tomatoes, potatoes, and green leafy vegetables are excellent sources of vitamin C. Although most vitamin C is completely absorbed in the small intestine, the percentage of absorbed vitamin C decreases as intraluminal concentrations increase.

It is a potent antioxidant and a cofactor for a family of biosynthetic and gene regulatory enzymes. It is an essential co-factor for collagen biosynthesis, carnitine and catecholamine metabolism, and dietary iron absorption.

Moreover, vitamin C contributes to immune defense by supporting various cellular functions of both the innate and adaptive immune systems. Vitamin C accumulates in phagocytic cells, such as neutrophils, and can enhance chemotaxis, phagocytosis, generation of reactive oxygen species, and ultimately microbial killing [67]. It is also needed for apoptosis and clearance of the used neutrophils from sites of infection by macrophages, thereby decreasing necrosis/NETosis and potential tissue damage. The role of vitamin C in lymphocytes is less clear, but it has been shown to enhance differentiation and proliferation of B- and T-cells, likely due to its gene-regulating effects [68].

#### The role of vitamin C for female gamete competence

Concerning female fertility, vitamin C can effectively scavenge ROS in female gametes. Among the most important impairments for the oocytes, ROS overproduction and DNA-related damages play certainly a key role in the cell’s homeostasis. In fact, ROS imbalance affects a large amount of physiological processes including oocyte maturation and development, placing vitamin C as a crucial antioxidant compound both for healthy fertility and age-related disfunction associated with infertility.

The vitamin C activity, in association with other immunonutrients, such as vitamin E or antioxidant-related enzymes (i.e., selenium in GPx enzymes), starts in the extracellular environment, and blocking the metal ions related oxidative stress can prevent lipid peroxidation. Subsequently, mechanisms such as direct repair through reduction of oxidized molecules could prevent DNA strand damage [47].

Moreover, several clinical trials investigated the human tolerance and the effectiveness on the oocytes quality, number of follicles, and estradiol levels through vitamin C supplementation, leading to an improvement of fertility, especially in PCOS-related infertility [38].

#### The role of vitamin C for male gamete competence

Regarding male fertility, high concentrations of vitamin C related with the human sperm is closely associated with the ROS scavenging of this immunonutrient. In fact, the intense mitochondrial activity leads to an increased production of ROS, potentially dangerous for the nucleic acids and for the lipid bilayer of spermatozoa, mostly rich in polyunsaturated fatty acids, and vitamin C is proposed as a powerful immunomodulator of male fertility regarding his antioxidant activity. Furthermore, a combination with vitamin E has been proposed for ROS scavenging action in human sperm [1].

### Vitamin D

Vitamin D (VD) is a fat-soluble vitamin present in few foods (oil fish, such as sardines, tuna, salmon, cod liver oil, egg yolks, shiitake mushrooms, liver, and organ meats), found in two major forms, D_2_ (ergocalciferol) and D_3_ (cholecalciferol), respectively arising from the plants’ sources and the animal tissue, but is also synthesized from dermal exposure after ultraviolet radiation (UVB, wavelength 290–315 nm) from its precursor, 7-Dehydrocholesterol – 7-DHC, called also provitamin D_3_). The UVB exposure of provitamin D_3_ in the skin breaks the B-ring to form previtamin D_3_, which undergoes thermally induced rearrangement (isomerization) to vitamin D_3_ [69].

Both vitamin D_2_ (from the plant sources) and vitamin D_3_ (from the animal tissue) are biologically inactive. They need further enzymatic conversion to their active forms. After hydroxylation at carbon 25 producing 25-hydroxyvitamin D, 25(OH)D3 (with a half-life of 2–3 weeks), it is transported to the kidney, where it is hydroxylated by 1α-hydroxylase (CYP27B1) at the carbon 1 of the A ring, producing 1,25-dihydroxy-vitamin D-1,25(OH)2D3, the active form of VD, with a half-life of 4–6 h. This process is driven by parathyroid hormone (PTH) and other mediators, including hypophosphatemia, and growth hormone (GH). [69, 70].

UVB is more prevalent during the hours from 10 am to 3 pm, and during spring, summer, and autumn 10–15 min of sun exposure (in this time slot) can produce adequate VD in light-skinned population; however, epidermal melanin of dark-skinned population can shield UVB radiation leading a lower production of vitamin D. Furthermore, cutaneous VD synthesis depends on the surface of skin exposed and sun exposure. Individuals such as vegans or people with an unbalanced diet are often associated with a VD deficiency, as well as patients with chronic diseases that involve intestinal malabsorption or liver/renal insufficiency [69, 71].

For these reasons, and for other conditions or diseases such as rickets during infancy and early childhood, osteopenia, or osteoporosis in adulthood, supplementation may be recommended after validating the VD deficiency [69].

Its most important biological role is promoting intestinal calcium absorption, facilitating calcium homeostasis, and promoting enterocyte differentiation. During hypocalcemia, the plasma level of ionized calcium falls and is detected by parathyroid gland calcium receptors. In response to these changes, PTH is secreted by the parathyroid gland, which stimulates 1-alpha-hydroxylation in kidneys to make more 1,25(OH)2D3. The elevation of calcitriol increases calcium transport within intestines, bones, and kidneys and regulates the osteoblasts and osteoclasts’ activity. When the plasma level of calcium rises back to normal, further PTH secretion decreases [69, 70].

The physiological interplay between VD, PTH and calcium levels demonstrates that adequate levels of 25(OH)D3 (calcidiol) is essential to improve the balance of plasma calcium levels. VD deficiency may result in inadequate circulating calcidiol levels, which decreases calcitriol synthesis and calcium absorption, leading to an increase of PTH levels, a condition known as secondary hyperparathyroidism [69, 70].

If its role in calcium metabolism and bone health is undisputed, its role in immune function, long-term health, and fertility represents a new frontier of medicine.

CYP27B1 (the limited enzymatic step for the VD activation) is also present in extrarenal sites, such as macrophages, osteoblasts, epithelial, endocrine and testis cells, confirming the new roles for this vitamin [71, 72].

Regarding the association between VD and the immune system, it affects the immune function in two ways:

Upregulation of the innate immune system and immunomodulation of the adaptative immune system [72].

Focusing on the innate immune system first, a major mechanism of VD action is via an endogenous antimicrobial peptide called cathelicidin (LL-37), which is generated in response to microbial invasion through activation of toll-2 like receptors (TLR-2) on monocytes and macrophages. Linking this process, the VD receptor element (VDRE) is interestingly contained in the promoter region of the gene for LL-37. VDRE is found only in the LL-37 gene promoters of primates, suggesting that the ability of VD to promote LL-37 antibacterial action is a relatively recent event in evolution [71, 72].

Converging on the signaling pathways, VD mediated his genomic actions through the estrogen receptor 1 (ESR1), estrogen receptor 2 (ESR2), and vitamin D receptor (VDR), but it also can be involved in non-genomic effects on male fertility.

#### The role of vitamin D for female gamete competence

VDR is also expressed in human ovarian and placenta tissue, and calcitriol turn-in is an essential element at the ovarian level for the steroidogenesis, for the respective biosynthesis of progesterone, estradiol, and estrone. The calcitriol action could be related to a decreasing level of anti-Mullerian hormone (AMH) mRNA and subsequently with overexpression of FSH receptor gene, indicating a positive mechanism VD dependent in follicular development and selection. VD can be classified as an ovarian reserve marker, especially with the AMH level combination, considering the presence of VDREs in the AMH promoter gene.

Several studies investigated the relationship between the immunomodulatory effect of VD treatment and cytokine production (primarily IL-6, IL-8, IL-10) on the endometrial cells of women with repeated implantation failure (RIF), often caused by over inflammation process, and VD can mediate its anti-inflammatory action facilitating the implantation process.

Moreover, many studies indicate a powerful relationship between VD and polycystic ovary syndrome (PCOS), mostly related to insulin resistance (IR), altered estrogen/androgen ratio, and obesity. These alterations are frequently BMI dependent and can be leading to follicular failure and anovulatory cycles, typically associated with PCOS, confirming the relationship with typical hyperandrogenism PCOS dependent and female infertility.

Furthermore, IR gene promoters contain VDREs, also confirming the importance of VD as an indirect ROS scavenger too, acting as a genomic master-switch ameliorating the insulin response and consequently the inflammation and redox status.

However, adequate VD serological levels are often associated with a decreased free androgen index (FAI), DHEA-S, LH/FSH ratio, and an increase of SHGB levels, promoting restoration of female fertility [41].

Novel studies focused on the role of VD among women with endometriosis. In this group of infertile women, 25–30% have endometriosis and 30–50% of women with endometriosis are infertile. The biological mechanisms linking endometriosis and infertility are distorted pelvic anatomy, altered peritoneal function, ovulatory abnormalities, and impaired implantation. The scientific literature elects as a first actor among these events the decidualization, a crucial step in the process of blastocyst implantation, usually impaired in the women with endometriosis, and VD plays a crucial impact on this process. Patients with vitamin D deficiency have to be found with a defective decidualization [54, 55].

The significant role of VD in endometriosis-associated infertility, menstrual cycle phases, and pregnancy is displayed by the physiology of the human endometrium, which is a steroid hormone-dependent tissue with a complex cellular regulation mediated by nuclear receptors [55].

Stromal endometrium cells were shown to express VDR and the active form of 1α-hydroxylase gene and protein, upregulated in early pregnancy versus the cycling endometrium phase [55].

In conclusion, several studies indicated the association between maternal VD deficiency and increased risk of pre-eclampsia, gestational diabetes mellitus, preterm birth, small-for-gestational-age infants, and impaired fetal bone formation. It's clear that VDR and 1αhydroxylase are active in reproductive tissues, but VDR was expressed differentially throughout pregnancy in placental, decidual, and ovarian follicular tissue supporting the hypothesis that VD is integrally involved in the physiologic changes of pregnancy [54, 72].

#### The role of vitamin D for male gamete competence

The interplay between VD, its receptor in male gonads, and the estrogen receptor (ESR1 and ESR2) suggest a key role for VD in male fertility as a co-master switch in the gene expression [54, 55]. In fact, ESR1 and ESR2 are present in Leydig, Sertoli, and germ cells in testis and spermatozoa. Typically, estrogens produce genomic effects after their binding to the nuclear receptors ESR1 or ESR2, but these hormones can produce a wide range of functions also with their binding to the plasma membrane receptors through different signaling pathways. Furthermore, the different expressions of ESR1 and ESR2 can be found in different types of cells. Recent *in situ* hybridization and immunohistochemistry experiments confirmed the mRNA presence and the following protein expression for ESR1/ESR2 in germ cells, ESR1 in interstitial cells, and ESR2 in Sertoli cells. This evidence suggests the dissimilar roles for VDR, ESR1, ESR2, and VDR/ESR1-ESR2 complex in spermatogenesis. Several different expressions have been found also in the human spermatozoa regarding VDR, ESR1, and ESR2. In particular, VDR has been found in the head and midpiece, while ESR1 is present in the equatorial segment and midpiece, whereas ESR2 can be found in the midpiece and the spermatozoa tail combined with ESR1 and VDR.

Confirming their role and examining their effects and signaling pathways, the strongest proofs arise from *in vivo* studies. ESR1/KO and ESR2/ knockout (KO) mice are infertile and show an increased rate of apoptosis at the early spermatogenic stages. At the cytological level, several sections report hyperplasia and hypertrophy for the Leydig cells, followed by morphological changes in the Sertoli cells.

After pharmacological low-dose treatments with estrogens, some authors confirmed that the induction of spermatogenesis via neuroendocrine mechanism ESR1 dependent involving an increase of follicle*-*stimulating hormone (FSH) levels, while a high-dose treatment leads to tubular atrophy, disruption of testicular structure, and apoptosis of germ cells. This evidence confirmed spermatogenesis as a hormone (estrogens) balanced process. Knowing that effects of estradiol on the different forms of receptors (ESR1 and ESR2) some studies were conducted with some estrogen receptor subtype-specific ligands, confirming distinct roles for ESR1 and ESR1. Respectively, ESR1 is more involved in the differentiation process and apoptosis whereas ESR2 is more competent in spermiogenesis regulation.

Moreover, acrosome development is an estrogen-dependent process: in fact, high levels of Aromatase can be found on the golgi complex of developing spermatid, and chromatin condensation (through histone displacement) related to spermatogenesis is also regulated by estrogen levels.

Many other VD effects are related to VDR signaling pathways. Originally identified as a chromatin-associated protein, VDR binds 1,25-D3 with high affinity and specificity and is associated with 1,25-D3 classical effects. VDR belongs to the steroid receptor family which includes receptors for retinoic acid, thyroid hormone, sex hormones, and adrenal steroids. VDR functions as an obligate heterodimer with Retinoid X receptors (RXR) for activation of VD target genes. The genomic mechanism of 1,25-D3 action involves the direct binding of 1,25(OH)_2_D_3_ activated VDR/RXR to specific DNA sequences (VD response elements – VDREs).

Several experiments indicated the presence of VDR in several male tissues, such as Sertoli cells, the main target of 1,25-D3, Leydig cells, seminiferous tubules, and caput epididymis.

At the spermatozoa levels, VDR can be found in the post-acrosomal part of the head, midpiece, and neck region.

As well as for ESR1 and ESR2, VDR/KO mice (from *in vivo* studies) are infertile. If VD deficiency mice has also a 45% reduction of fertility, on the other hand, 1,25-D3 can promote expression of CYP19 (encoding gene for Aromatase), confirming the necessary interplay between VD, VDR, estrogen actions, and male gonad functions.

In addition, an adequate tissue level of 1,25-D3 can lead to increased sperm survival, is essential for testicular maturation, and can promote the acrosomal reaction. Furthermore, 1,25-D3 through VDR can increase intracellular calcium (Ca^2+^) levels, fundamental for spermatozoa motility.

Concluding, an interesting positive feedback mechanism of regulation for the male gamete competence could exist. 1,25-D3 can bind VDR localized at the plasma membrane or intracellular VDR and could regulate CYP19 and ESR1 gene expression, Aromatase activity, and estradiol production. Estradiol can regulate VDR gene expression by ESR2 localized at the plasma membrane and CYP19 gene expression, confirming the novel proposal role of VD in the hormone balance and male fertility [42].

Regarding human clinical evidence, there is a significant reduction in sperm motility in individuals with low serological levels of VD (34% of mobile spermatozoa/total spermatozoa compared with the control group). Furthermore, an increased rate of morphological alterations in the VD deficiency group can be found compared to the control group.

On the other hand, a high dose supplementation of VD (300,000 I.U. in a single dose), followed by a low dose of VD (1.400 I.U. every day, for 180 days) can significantly improve the rate of fertility in VD deficiency men. Men with oligoasthenozoospermia treated through a low dose of VD associated with Vitamin E and Ca^2+^ show a statistically significant gain of function compared with the control group.

Concluding, a daily VD supplementation can modulate the hormone axis: some evidence from clinical trial demonstrate a rise of free and total testosterone levels, such as for the correlation between VD concentrations and sperm motility/morphology improvement and improved semen quality and pregnancy rates in VD deficiency men [56].

### Vitamin E

Vitamin E, or tocopherol, is a fat-soluble antioxidant contained in vegetable oils, wheat germ, cereals, meat, poultry, eggs, dairy products, fruit, and vegetables. The recommended daily dose of vitamin E is 200-400 mg. It has the function of protecting the cell membrane from ROS and neutralizing them as well as preventing lipid peroxidation.

Vitamin E appears to be involved in innate and adaptive immunity. It would seem to maintain or improve the cytotoxic activity of NK cells and reduce the production of prostaglandin E2 by inhibiting the activity of cyclooxygenase-2. Vitamin E improves synapse formation in naive T cells and has a modulatory action on the balance between Th1 and Th2 [35].

The mechanism of action of vitamin E is not completely understood. However, its role as an antioxidant allows the maintenance of a steady state, reducing peroxyl and alkoxy radicals. In the redox reactions in which it is involved, tocopherol can regenerate itself via ascorbic acid or reduced glutathione. This shows its synergy with other antioxidant systems [54].

#### The role of vitamin E for female gamete competence

Regarding female fertility, ROS are known to influence various physiological functions including oocyte maturation, ovarian steroidogenesis, ovulation, implantation, blastocyst formation, luteolysis, and luteal maintenance in pregnancy [54].

In addition, excessive oxidative stress has been shown to damage oocytes during maturation and cleavage, leading to chromosome segregation during meiosis, and an impaired fertilization process. ROS-mediated damage contributes to an imbalance in microtubule assembly and cytoskeleton alterations in oocytes [73].

Vitamin E has been shown to regulate ROS production probably because it readily crosses the placenta. Under normal circumstances, antioxidants actively remove ROS, but when the production of ROS exceeds the rate at which they can be counteracted by antioxidants, we talk about oxidative stress and this produces a great impact on cellular function.

The increase in vitamin E levels in pregnant women agrees with a higher probability of finding normal oocytes and more pregnancies through in vitro fertilization by reducing ROS.

This is consistent with its role as an antioxidant, protecting oocytes from ROS and preventing lipoperoxidation.

Due to the limited literature regarding vitamin E functionality in human reproduction, further studies are needed to clarify its role in female gametogenesis [73].

#### The role of vitamin E for male gamete competence

It was shown that in infertile men treated with 1g of vitamin E and 1g of vitamin C they reduced the level of DNA damage in the intervention group, although there was no significant relationship between vitamins and the parameters of motility and concentration of spermatozoa. The high levels of sperm DNA damage are correlated with a higher infertility rate. The same treatment continued for 2 months improved the success rate of Intra-Cytoplasmic Sperm Injection (ICSI) [27].

While the daily supplementation of selenium (200 μg) with vitamin E (400 IU) for at least 100 days resulted in a 52.6% improvement in spermatozoa motility and morphology and 10.8% spontaneous pregnancy compared to control [27].

It would therefore seem that the infertility rate decreases following the intake of immunonutrients. Mainly ROS damage appears to play a role in 30–80% of subfertile men. The use of antioxidants, particularly vitamin E supplementation, is useful in the treatment of infertility in males with excess ROS [27]. A direct relationship was found between vitamin E levels in seminal plasma and the percentage of motile spermatozoa in semen [28].

In the literature, it is reported that the synergy of vitamin E and selenium led to significant increases in motility and activity of glutathione peroxidase in seminal plasma, improved sperm motility, and lipid peroxidation markers. Furthermore, they showed a spontaneous pregnancy rate of 21% in the treatment group compared to the total absence of pregnancies in the placebo group [28].

Although the supplementation of antioxidants in various combinations would seem to reduce the rate of infertility, the necessary and sufficient dose to counteract OS is not known.

This involves supplementation with non-standard doses. For instance, vitamin E at high doses has been shown to increase mortality in patients [57].

Recent studies demonstrate an increased risk of cardiovascular complications, especially heart failure, and all-cause mortality at doses greater than 400 IU/day. Doses greater than 800 IU/d increase antiplatelet effects and bleeding risks and have been found to interact with the effects of warfarin. Vitamin E intake can block the conversion of vitamin K [34].

The OS can influence the clinical outcome of Assisted Reproductive Technology (ART). ROS in seminal plasma has been found to have a significant negative correlation with fertilization rate in both In Vitro Fertilization (IVF) and ICSI. However, few studies have examined the effect of antioxidant therapy on birth rates both naturally and after ART.

Vitamin E supplementation had a beneficial effect on in vitro sperm function and fertilization rates via IVF compared to placebo groups. Vitamin E (200 mg per day) has been found to reduce malondialdehyde levels (an indicator of lipid peroxidation) and improve fertilization rates with IVF [58].

In recent years, screening for OS is being done in the evaluation of infertile men. Assessment of seminal OS levels would help monitor antioxidant therapies and establish effective doses and durations. However, the availability of the tests, the complexity, the cost-effectiveness, and the lack of a standardized method of analysis are among the disadvantages that prevent their routine use in clinical practice [58].

Antioxidants generally have a favorable effect on male fertility. However, further studies are needed to identify the optimal antioxidant regimen that can be used safely and efficiently in clinical practice.

### Omega 3 fatty acids

Omega 3 fatty acids are polyunsaturated fatty acids also called n-3 fatty acids or omega-3 fatty acids, composing a heterogeneous group of fatty acids with a double bond between the third and fourth carbon atoms from the methyl end, an interesting characteristic able to directly remove the ROS species. This group includes linolenic acid (ALA) consumed from various plant sources and eicosapentaenoic acid (EPA) and docosahexaenoic acid (DHA), derived mostly from fish, remembering the recommended daily intake of omega-3, which is 1000-1500 mg/day [35].

Among the fatty acids, omega-3 fatty acids have the most potent immunomodulatory activities, and among the omega-3 fatty acids, EPA and DHA are biologically stronger than ALA. Some of the effects of omega-3 fatty acids include actions upon intracellular signaling pathways, transcription factor activity, and gene expression.

Generally, ALA, DHA, and EPA exert an inhibitory effect on the activation of immune cells from both the innate and the adaptive branches. Interestingly, some specific immune functions are promoted by dietary omega-3 fatty acids in specific immune cell types (i.e., phagocytosis by macrophages and neutrophils or Treg differentiation), suggesting that omega-3 fatty acids do not act as nonspecific immune-repressors.

Furthermore, they exert anti-inflammatory and antioxidant properties that can support the immune system. In particular, EPA and DHA are enzymatically converted to specialized pro-resolving mediators (SPMs) known as resolvins, protectins, and maresins, and their functions can orchestrate the resolution of inflammation.

These molecules can orchestrate alongside others the balance of the intracellular redox status, supporting the immune system and mitigating the adverse effects of inflammation. The inflammatory response is fundamental to immunity mechanisms. DHA and EPA, including resolvins, protectins, and maresins are involved in regulating inflammatory processes and responses. As a result, they support the resolution of inflammation and consequently support healing, which may be delayed in individuals with deficiencies of DHA and EPA [74].

#### The role of omega-3 fatty acids for female gamete competence

Omega-3 fatty acids can directly or indirectly impact female infertility. Omega-3 fatty acids can ameliorate the oocyte quality and embryo implantation, whereas dietary fatty acids intake is mostly involved in insulin resistance (IR), affecting negatively ovulatory function. Women with a poor intake of dietary omega-3 fatty acids had lower fecundability than women with an omega-3 rich diet [59].

Regarding PCOS, the primary outcome proposed for the omega-3 fatty acids includes the change on the homeostatic model assessment (HOMA) index of IR, because these fatty acids can decrease the biosynthesis of prostaglandins through competitive inhibition of Cyclooxygenase-2 (COX-2). This mechanism leads to lower production of inflammatory cytokines, such as IL-6 and TNF-α, which can promote the M1 macrophage recruitment and subsequently the adipose tissue inflammation, primary cause of type 2 diabetes mellitus (T2DM) or IR. Furthermore, omega-3 fatty acids have a particular skill for the alteration on M1 macrophage polarization, acting as a secondary ROS scavenger too and blocking the oxidative process and inflammatory status. Meanwhile, omega-3 is not directly related to the FSH, LH, and estrogen levels, since PCOS is closely associated with insulin resistance and hyperandrogenism omega-3 fatty acid may be recommended for the treatment of PCOS and PCOS related female infertility [46].

#### The role of omega-3 fatty acids for male gamete competence

Regarding male infertility, the primary role of omega-3 fatty acids arises from their concentration in spermatozoa testis. Even more than neuronal cells, spermatozoa heads show the most prominent concentration of omega-3 in their lipid membranes.

Omega-3 accumulate in testis through two different ways: the first, through a passive diffusion mediated by the membrane lipid bilayer, and the second through a CD36 glycoprotein related to transport in Sertoli cells, which are rich in omega-3 fatty acids, mostly used for germ cells lipid remodeling at the membrane level, a key element for their proper development [43, 44].

Furthermore, the Sertoli cells’ capacities for desaturation and elongation are essential for the maintenance of spermatozoa membrane integrity and function. In addition, several hormones can modify the lipid characteristic of germ cells membrane through Sertoli cells receptors and their relative pathways. In particular, Adrenocorticotropic hormone (ACTH) can lead to an increase of omega-6 biosynthesis, while LH and Testosterone drive towards an increase of omega-3 membrane concentration maintenance. This evidence makes of an interesting scenario for the relationship between omega3 importance and dietary omega3/omega6 ratio in male fertility, especially in BMI-related syndromes, such as male obesity secondary hypogonadism (MOSH syndrome). Precisely for the MOSH syndrome, an omega-3 treatment has been proposed for the amelioration of related symptoms [45].

The spermatozoa membrane storage capacity of omega-3 fatty acids is essential also for the correct acrosome biosynthesis and related reaction, a key element for natural male fertility.

Furthermore, the directly omega-3 fatty acids action is against ROS activity, especially related to lipid membrane peroxidation or secondary mostly resulting by a ROS spread born from the lipid bilayer oxidation and direct to DNA damage. High DNA fragmentation index related to low-sperm quality is often associated with lower DHA levels in spermatozoa. Moreover, several pieces of evidence indicate Peroxisome proliferator-activated receptor gamma (PPAR-γ) as a possible target for omega-3 fatty acids stimulation, through gene overexpression and enzymatic potential changes (allosteric secondary domain in PPAR-γ). Indeed, PPAR-γ over omega-3 activation can improve the spermatozoa assembly and mediate antiapoptotic effects through its signaling pathways, minimizing oxidative stress and inflammatory process. Omega-3 induced PPAR-γ activation can block NF-κB’s activity, resulting in a decrease in inflammation and DNA damage.

In addition, a negative correlation between dietary trans fatty acids and PPAR-γ gene expression is widely demonstrated.

The direct and secondary anti-ROS acts, and generally the antioxidant activity of omega-3 fatty acids against the aging-related condition that generally targets the spermatozoa integrity, is another proof for the key role of these immunonutrients.

Concluding, the typical spermatozoa lipid membrane changes, their membrane protein/ion channels functions, and the physiological maintenance suggest a putative mechanism for omega-3 fatty acids on male fertility and spermatozoa balanced activity [44].

### Selenium

Selenium is a trace element essential for human reproduction. It was shown that women with selenium concentrations <0.95 micromol/L took longer to conceive, while levels below 45 μg/L are considered dangerous [60].

Brazilian nuts, cereals, beef meat, seafood, and eggs are sources rich in selenium and the recommended daily dose is 50–400 mcg [1].

The recommended dietary intake of selenium is equal to the amount of selenium needed to maximize the synthesis of glutathione peroxidase [39].

Selenium also plays an important role in the immune response. It can modulate the host defense system (influencing leukocyte and NK cells). Selenium is also involved in the production of immunoglobulins and increases the production of IFN-γ [35].

Selenoproteins play an important role in signaling, lipid biosynthesis, cell cycle, calcium regulation, and ultimately protein folding [39].

It plays a fundamental role as an antioxidant in the cell cycle and immune function through selenoproteins. The antioxidant defense system consists of enzymes and non-enzymatic components. Some of these enzymes containing selenium as an essential cofactor are glutathione peroxidase, catalase, superoxide dismutase, and thioredoxin reductase, essential for the scavenging action from the lipid bilayer to the DNA damage ROS related.

#### The role of selenium for female gamete competence

In 1995, evident traces of selenium in human follicular fluid and manifestations of selenium-dependent enzymatic activity were reported for the first time. It was noted that patients with idiopathic infertility had significantly reduced follicular selenium levels compared to those with known causes of infertility. Based on findings, it was deduced that the antioxidant activity of the glutathione peroxidase (GPX) selenoenzyme in the microenvironment of the follicles may play a significant role in gametogenesis and fertilization [61].

The relationship between selenium and female fertility has not been extensively studied, and the few studies present focus on the role of selenium during pregnancy and not in earlier stages.

Despite this, a strong association was found between glutathione peroxidase1 (GPX1) and the dominant follicle. Probably its antioxidant action against ROS allows the development and protection of the dominant follicle [75].

The relationship between female fertility and selenium has been demonstrated in a few studies which, in general, have generally observed relatively low serum selenium and follicular fluid levels to be linked to increased infertility.

Many researchers demonstrated a marked reduction in the expression of GPX1 in selenium deficiency highlighting the possibility that ovarian pathologies can be improved through its integration.

Despite sparse information on the relationship between female sex hormones and selenium, researches conducted with healthy women point to a correlation between estrogen content and selenium content as well as GPX activity depending on the phase of the menstruation cycle.

Its role in this process remains unknown. It has been observed that the first meiotic division in these cells is induced by an increase in the concentration of ROS and that it is inhibited by antioxidants. This allows us to assume that ROS secretion, regulated by a pre-ovulatory ovarian follicle, is a significant promoter during ovulation [62].

Regarding ART, it has been shown that selenium supplementation can improve the outcome of in vitro fertilization. Selenium concentrations in follicular fluid have been suggested as a marker of infertility, even if the evidence is limited [40].

#### The role of selenium for male gamete competence

In men, particularly in the testicles, high concentrations of selenium are present, essential for normal testicular development, spermatogenesis, motility, and vitality of spermatozoa.

Several studies have shown that two selenoproteins are required, phospholipid-hydroperoxide glutathione peroxidase 4 (GPX4) and selenoprotein P for normal spermatogenesis. Furthermore, selenoproteins are involved in maintaining the structural integrity of sperm and organizing chromatin [40].

It seems that GPX4 has, in addition to an antioxidant role, a structural function. During spermatogenesis it protects against oxidative damage, while in the next stage, it becomes a component of the mitochondrial sheath, providing stability and motility to the spermatozoa.

Selenium is present in organic and inorganic forms. Organic forms have a greater bioavailability, they are assimilated more efficiently and could be rapidly used for the synthesis of selenoproteins under stressful conditions.

It is important to underline that, in conditions of insufficient or low selenium intake, the synthesis of some selenoproteins such as GPX4 takes priority over the others [63].

The lack of selenium leads to the loss of motility in the spermatozoa and an increase in the incidence of anomalies, mostly of the heads. Several studies have shown a correlation between selenium levels in seminal plasma and the quality of normal sperm in a sample. However, these results have not been confirmed in other trials [1].

Moreover, selenium deficiency can lead to the NF-kB and a downregulation of p65 and p60, promoting the inflammation pathways and immune response. Indeed, these activations are related to a downregulation of ERK signaling and totalize the inflammatory response leading to DNA damage, counteracted by Se-dependent antioxidants enzymes.

Furthermore, selenium also plays a role in the biosynthesis of testosterone. The concentrations of testosterone in the blood would have a positive correlation with the concentrations of selenium. However, the underlying mechanisms are still unclear [63].

Typically, selenium has been studied in combination with other vitamins; particularly vitamin E as their synergism as antioxidants is well known [28].

A meta-analysis of observational studies evaluated the effect of certain antioxidants (including selenium and vitamins) on male infertility and showed that some of them are associated with sperm quality. However, no conclusions were drawn regarding the intake of selenium [76].

There are several combinations used between selenium and other vitamins (e.g., selenium 100–300 μg / day or 200 μg of selenium plus 600 mg of N-acetylcysteine through oral supplementation per day). All interventions had favorable outcomes in all seminal parameters. Interestingly, supplementation improved sperm concentration, motility, and morphology [27].

Indeed, selenium deficiency has been related to spermatogenic failure, testicular hypotrophy, atrophy of the seminiferous epithelium, abnormal sperm motility and morphology. The combined administration for 100 days of selenium (200 μg/day) and vitamin E (400 UI/day) resulted in a higher spontaneous pregnancy rate in treated patients than in controls [58].

However, in the literature, some studies do not show any improvement in concentration, morphology, and motility despite the increase in serum and seminal levels of selenium.

Baseline semen analysis has been criticized for being a poor predictor of fertility.

It does not provide information on the sperm's potential to fertilize an ovum or the maturation processes required to achieve fertilization. Therefore, advanced tests were developed to improve the predictive power of sperm studies. Of the several advanced sperm function tests that have been developed, SDF and OS measures have been the most studied [58].

### Zinc

Besides iron, zinc is the most abundant metal in our bodies. It is an important component of zinc finger proteins (ZFPs), one of the most common classes of transcription factors involved in numerous growth and development processes: DNA binding, RNA packaging, activation of transcriptional and translational factors, apoptosis regulation, and protein folding [29]. Despite its importance, however, about one-third of the population lacks it [29]. Zinc deficiency can compromise the immune system on several fronts as it can affect the activity of phagocytic and natural killer cells as well as that of T-cells. Similarly, it has been shown that restoring adequate zinc levels can resolve certain immune imbalances and reduce the frequency of infections [33].

As the human body hasn’t developed any specialized zinc storage system, it is important to ensure a daily intake of 11–40 mcg [1]. In particular, the European Food Safety Authority recommends an intake of 7.5–12–7 mg/day in adult women, with an increase of 1.3 mg/day if pregnant [65]. Wheat, seeds, and beef products are rich in this trace element.

#### The role of zinc for female gamete competence

Serum zinc levels decrease physiologically during pregnancy, due to hormonal changes, hemodilution, increased excretion of zinc, and its transfer to tissues and the fetus. However, a marked deficiency of this element may increase the rate of fetal malformations, IUGR, and long-term complications [77].

Zinc supplementation alone or in combination with other elements appears to reduce the rate of preterm birth by 14% in women with poor nutritional status [78].

Proper zinc homeostasis is crucial in oocyte development. This cofactor is involved in the inactivation of the proto-oncogene serine/threonine-protein kinase mos (Mos)/mitogen-activated protein kinase (Mapk) pathway and in the modulation of cell division cycle 25 phosphatases (Cdc25) and early meiosis inhibitor 2 (Emi2), thus intervening in both prophase I arrest and the subsequent progression of egg cell maturation [79].

At the oocyte level, zinc is also essential as a cofactor of CCCTC-binding factor (CTCF), a multifunctional molecule involved in the spatial organization of chromatin. A functional deficiency of this molecule modifies the gene expression of the egg cell and affects its maturation and embryonic development [80].

#### The role of zinc for male gamete competence

Seminal plasma zinc concentrations differ significantly between fertile and sub-fertile men. Zinc seems to protect sperm structure: in addition to limiting chromosomal and infection-related damage, it plays an important role in the development and maturation of spermatozoa [27].

Zinc plays a critical role in germ cell survival, spermiogenesis, and epigenetic regulation of spermatogenesis, and its presence in prostate secretions stabilizes sperm before ejaculation. The importance of its role in these processes is shown by the fact that in azoospermic, oligozoospermic, and asthenozoospermic patients reduced levels of zinc can be found [29].

Zinc-deficient patients’ spermatozoa show flagella abnormalities such as hypertrophy/hyperplasia of the fibrous sheath, axonemal disruption, or abnormal axonemal structure, caused by the defects of the inner dynein arms [1, 28, 34].

Moreover, reduced levels of zinc are associated with a low rate of sperm fertilization [43].

Serum changes associated with zinc deficiency also include an increase in Tumor Necrosis Factor-alpha (TNF-a) and pro-inflammatory cytokines and a decrease in anti-inflammatory cytokines such as interleukin-2 (IL-2) and interleukin-4 (IL-4). The resulting inflammatory state increases the recruitment of leucocytes to the reproductive system, leading to an increase in free radicals and alterations in the individual's reproductive capacity [29].

Zinc also influences the body's oxidative state directly, both antagonizing reactive metals such as iron and copper and binding proteins sulfhydryl groups [58, 64].

Zinc deficiency and subsequent oxidative stress have been associated with Leydig cell damage/apoptosis, defects in luteinizing hormone (LH) receptor function, and altered steroid hormone synthesis. It should not be forgotten that many steroid hormone receptors have zinc fingers in their structure. Thus, a zinc deficiency may be associated with a deficit in testosterone function, resulting in hypogonadism, inhibition of spermatogenesis, and infertility [29].

Other evidence supporting the importance of the role that zinc has at the testicular level is the improvement in sperm concentration, motility, and integrity and the increased pregnancy rate in sub-fertile males supplemented with it [1, 58]. The 26-week administration of 5 mg of folic acid and 66 mg of zinc has been shown to improve sperm concentration, while the daily supplementation of 220 mg of zinc sulfate positively correlates with semen volume and motility rate [27].

However, other studies failed to demonstrate the effect of a 16 weeks supplementation with folic acid and zinc, individually and combined, on sperm morphology and motility [27].

Focusing on the antioxidant activity, zinc supplementation in infertile males restores peroxynitrite levels and arginase and Nitric oxide (NO) synthetase activity to the values found in fertile patients [27].

OS and SDF and apoptosis appear to be reduced in patients receiving zinc supplementation, alone or in association with vitamin E and/or C. However, the power of this correlation is damaged by the small sample used to assess it [28].

### Microbiota

Regarding the fertility field, dietary patterns, and micronutrients activity, the emerging crosstalk between gut microbiota and human cells is becoming a new topic of scientific research. Gut microbiota is represented by approximately 1014 microbes, ranging about between 300 and 1000 different species, and can biosynthesize thousands of metabolites. Typically, the gut microflora is represented mainly by the phyla Bacteroidetes, Firmicutes, Actinobacteria, Proteobacteria, Fusobacteria, and Verrucobacteria.

The two phyla Firmicutes and Bacteroidetes represent 90% of gut microbiota. The Firmicutes phylum is characterized by more than 200 different genera (e.g., Lactobacillus, Bacillus, Clostridium, Enterococcus, and Ruminococcus). Bacteroidetes contain two predominant genera (e.g., Bacteroides and Prevotella. The Actinobacteria phylum is relatively less numerous and mainly represented by the Bifidobacteria [81].

The mutual interaction between diet, micronutrients, and gut microbiota can result in a homeostatic status or can cause dysbiosis. Dysbiosis is a biodiversity alteration of gut microbiota, promoting mechanisms of immune intolerance which culminates in topic and systemic inflammation. On the other hand, dietary patterns such as Mediterranean diet characterized by a higher intake of PUFAs (e.g., ω-3 FAs, olive oil), vegetables, fruits, and dried fruits, moderate consumption of fish and white meat and in correspondence with a lower use of red meat, is related to gut microbiota biodiversity balance, decrease of oxidative processes and inflammatory status, environmental factors associated to Cardiovascular diseases (CVDs) and chronic degenerative diseases (CDDs) [2, 82]. 

In particular, this mechanism is explained through the short-chain fatty acids (SCFAs) production related to gut microbiota health and from the associated degradation of plant-based foods (e.g., provided by Mediterranean diet adherence), meanwhile the typical “Western diet” dysbiosis results in a higher concentration of trimethylamine N-oxide (TMAO).

Derived from the fermentation of fibers and non-digestible carbohydrates, SCFAs such as acetate, butyrate, and propionate are fundamental biomolecules concerned with maintaining intestinal and inflammation/oxidative status homeostasis. Indeed, SCFAs are related to the upregulation of gene expression and transcription of genes involved in the maintenance of intestinal mucosal integrity [83].

Induced by the Mediterranean diet, the gut microbiota activity results in high production of SFCAs, associated with a lower inflammation status and decrease of ROS and their related processes. In this way, the diet-induced modulation of microbiota and NS can contribute to reducing the CDDs and CDVs risks.

A new topic of research can be suggested by estrobolome, the collection of human-related microbiota species and their estrogens reactions, mostly promoted by gut microbial β-glucuronidase (GUS) enzymes [84].

During phase II metabolism, human UDP-glucuronosyltransferase enzymes (UGTs) attach a glucuronic acid unit to a large amount of endo- and xenobiotics, inactivating in this way these molecules and marking them for excretion. GUS enzymes within specific gastro-intestinal (GI) sections can deconjugate glucuronic acid and reactivate some specific molecular targets, reversing phase II glucuronidation.

This biochemical reaction can include estrogen, typically glucuronidated in the liver during phase II metabolism. In fact, an estrobolome enriched in β-glucuronidase enzymes can promote estrogen metabolite deconjugation reactions may result in greater reabsorption of free estrogens and result in an increase of estrogen activity. In particular, the deconjugated estrogen can flow up the bloodstream and subsequently bind to the ERα and ERβ, stimulating gene activation and several intracellular signaling pathways [85].

Confirming this hypothesis, endometriosis is often matched with dysbiosis, typically characterized by a decrease of Lactobacillus spp. and an increase of Gram-negative spp. Dysbiosis can disrupt the estrogen balance altering the GUS enzymes related deconjugation processes.

Another typical disease associated with infertility and microbiota status is PCOS. PCOS and abdominal adiposity are closely connected and result in a vicious circle characterized by androgen excess and related alteration of estrogen levels. Androgen excess in fact favors the abdominal deposition of body fat, and visceral fat facilitates androgen excess of ovarian and/or adrenal origin, and with autocrine, paracrine, and endocrine signaling can promote the induction of IR and hyperinsulinism [21].

In this context, gut microbiota can promote metabolism and immune response, acting as one of the major leading actors supporting the PCOS therapies. On the other hand, gut microbiota dysbiosis can cause IR, which is strongly linked to the development of PCOS. In particular, several typical microbiota changes may occur in PCOS development and with clinical manifestations of PCOS.

PCOS in fact can be associated with a decrease in alpha (α) diversity (species diversity present within the GI tract) and a change in beta (β) diversity (species diversity between any two patches and their communities). Concluding the last vicious circle, changes in α and β diversities can contribute to the IBS development, change the androgen/estrogen balance, affect the insulin and glucose tolerance levels and improve the inflammation processes, often altered in PCOS patients, worsening the PCOS symptoms [86, 87].

Finally, even though for male infertility the microbiota investigation is still inadequate, a future therapeutic approach for PCOS/Endometriosis related infertility may involve probiotics, prebiotics, and synbiotics, as well as fecal microbiota transplantation [88].

Nevertheless, a long duration maintenance of healthy gut microbiota status is through the effects of nutrition, often in association with physical activity (PA), electing several dietary patterns such as Mediterranean diet as the most powerful co-player capable to preserve the microbial richness and diversity.

## Conclusion

Given the susceptibility of reproduction to inflammatory and oxidative processes, immunonutrients especially provided through healthy diets, could have a fundamental role in preserving the functional integrity of the male and female germline.

An optimal nutritional status guarantees a balance in human cells, counteracting many inflammatory and oxidative stress processes and orchestrating a powerful stream that leads to a better response in the fertility field. This concept translates into a high-quality diet, enriched with anti-inflammatory and immunomodulatory nutrients, including vitamins and minerals, with protective and improving characteristics for the germinal cells.

Many of the oxidative processes that carry on a malfunction in the germinal cell lines can be beheld by the biochemical structure of the vitamins examined in this review.

Moreover, the capacity of these nutrients to induce immunomodulation could be mediated indirectly through interaction with immune cells supporting the antioxidant action which can typically improve fertility.

However, scientific literature on immunonutrients applied to improving the competence of gametes is currently limited. This deficit should be addressed as soon as possible, by starting clinical studies aimed at identifying the best nutrients and defining their dosages, to preserve and support fertility.

## Abbreviations

7-DHC: 7-dehydrocholesterol
ACTH: adrenocorticotropic hormone
ALA: linolenic acid
ART: assisted reproductive technology
ATP: adenosine triphosphate
BMI: body mass index
C/EBP: CCAAT/enhancer-binding protein
Ca^2+^: calcium
CTCF: CCCTC-binding factor
Cdc25: Cell division cycle 25 phosphatases
CVDs: Cardiovascular diseases
CDD: Chronic degenerative diseases
DHA: Docosahexaenoic acid
EMI2: early meiosis inhibitor 2
EPA: eicosapentaenoic acid
ER: endoplasmic reticulum
ESR1: estrogen receptor 1
ESR2: estrogen receptor 2
FSH: follicle*-s*timulating hormone
GH: growth hormone
GI: gastro-intestinal
GPX: glutathione peroxidase
GPX1: glutathione peroxidase 1
GPX4: glutathione peroxidase 4
GUS: gut microbial β-glucuronidase
HOMA: homeostatic model assessment
ICSI: intra-cytoplasmic sperm injection
IFN: interferon
IL: interleukin
IR: insulin Resistance
IUGR: intrauterine growth restriction
IVF: in vitro fertilization
KO: knockout
LH: luteinizing hormone
LL-37: cathelicidin
MAPK: mitogen-activated protein kinase
MD: mitochondrial dysfunction
MOSH: male obesity secondary hypogonadism
MSG: monosodium glutamate
mTOR: mechanistic target of rapamycin
NK: natural killer
NO: nitric oxide
NS: nutritional status
OS: oxidative stress
PA: physical activity
PPAR-γ: peroxisome proliferator-activated receptor γ
PTH: parathyroid hormone
PCOS: polycystic ovary syndrome
ROS: reactive oxygen species
RXR: Retinoid X Receptors
SCFAs: short-chain fatty acids
SDF: sperm DNA fragmentation
SPF: sun protection factor
SPMs: specialized pro-resolving mediators
T2DM: type 2 diabetes mellitus
TLR-2: toll-2 like receptors
TMAO: trimethylamine N-oxide
TNF-α: tumor necrosis factor-alpha
UGTs: UDP-glucuronosyltransferase enzymes
U.I.: international unit
UVB: ultraviolet radiation
VD: vitamin D
VDR: vitamin D receptor
VDRE: vitamin D receptor element
VDREs: VD response elements
ZFPs: zinc finger proteins
